# Arthralgia and fever as dominant predictors of Chikungunya confirmation: an explainable artificial intelligence approach

**DOI:** 10.3389/fmed.2026.1814257

**Published:** 2026-04-13

**Authors:** Xinyu Lu, Dan Yin, Hanwen Zhang

**Affiliations:** 1 Health Management Research, Zhongshan Second People's Hospital, Zhongshan, Guangdong, China; 2 Department of Applied Mathematics & Statistics, Stony Brook University, Stony Brook, NY, United States

**Keywords:** arboviral disease classification, Chikungunya, clinical decision support, explainable artificial intelligence, SHAP

## Abstract

The clinical differentiation of Chikungunya from other acute febrile illnesses poses a significant diagnostic challenge due to the substantial symptom overlap among co-circulating arboviruses such as Dengue and Zika, particularly in resource-constrained settings where laboratory confirmation is frequently delayed or unavailable. This study proposes an interpretable machine learning framework for the prediction of laboratory-confirmed Chikungunya cases versus suspected cases that were subsequently discarded after laboratory investigation, using a large-scale, nationally representative dataset of 12,709 notification records derived from Brazil's Information System for Notifiable Diseases (SINAN), spanning 2013 to 2020 and encompassing 26 clinical symptom, comorbidity, and sociodemographic features. Four supervised learning algorithms—Logistic Regression, Random Forest, XGBoost, and LightGBM—were systematically evaluated under 5-fold stratified cross-validation, with Random Forest achieving the highest discriminative performance (AUC = 0.785 ± 0.005) and XGBoost demonstrating the best probability calibration reliability. Considering the trade-off between discrimination and calibration, XGBoost was identified as the most suitable model for potential clinical deployment. To ensure clinical transparency, a structured three-level SHapley Additive exPlanations (SHAP) interpretability analysis was conducted on both gradient boosting models, encompassing global feature importance ranking, feature-level directional and interaction effects, and local patient-level prediction decomposition. The SHAP analysis consistently identified arthralgia (mean |SHAP|: 0.7407 in XGBoost, 0.7489 in LightGBM) and fever (0.5043 and 0.4881, respectively) as the two dominant predictors of Chikungunya confirmation, followed by age, education level, and rash, while comorbidities contributed negligibly to case discrimination. Cross-model validation between XGBoost and LightGBM revealed highly concordant feature importance rankings and directional effect patterns, confirming that the identified clinical predictors are robust and independent of algorithmic choice. These findings demonstrate the practical value of combining machine learning with SHAP-based explainability for supporting clinical triage of suspected arboviral cases, providing a transparent, evidence-based diagnostic support tool that aligns data-driven insights with established clinical knowledge and enables personalized patient-level explanations at the point of care. Importantly, the model distinguishes confirmed Chikungunya from discarded suspected cases rather than from confirmed infections with other specific arboviruses, and its predictions should be interpreted within this operational context.

## 1 Introduction

Chikungunya is a mosquito-borne viral disease transmitted primarily by *Aedes aegypti* and *Aedes albopictus* vectors, characterized by acute febrile illness accompanied by debilitating polyarthralgia that can persist for months or even years after initial infection. Since its re-emergence in the Americas in 2013, Chikungunya has caused widespread epidemics across Latin America and the Caribbean, with Brazil bearing a disproportionately high burden due to its tropical climate, extensive urbanization, and favorable ecological conditions for vector proliferation (1, 2). The Brazilian Ministry of Health recorded millions of suspected arboviral disease notifications between 2013 and 2020 through the national Information System for Notifiable Diseases (SINAN), reflecting the enormous scale of the public health challenge posed by co-circulating arboviruses including Dengue, Chikungunya, and Zika (3). The clinical differentiation of Chikungunya from other arboviral infections remains a significant diagnostic challenge, as these diseases share a constellation of overlapping symptoms—fever, myalgia, headache, and rash—particularly during the acute phase of illness. Laboratory confirmation through serological, virological, or molecular testing represents the gold standard for definitive diagnosis; however, such testing is frequently unavailable, delayed, or cost-prohibitive in resource-constrained primary healthcare settings where the majority of suspected cases initially present (4). This diagnostic bottleneck results in delayed treatment initiation, suboptimal patient management, and impaired epidemiological surveillance, underscoring the urgent need for rapid, accessible, and reliable clinical decision support tools that can assist frontline clinicians in prioritizing patients for confirmatory testing and initiating appropriate management strategies based on readily available clinical and sociodemographic information (5).

The advent of machine learning (ML) has opened promising avenues for automated disease classification based on routinely collected clinical data, offering the potential to augment clinical judgment with data-driven predictive insights (6). Supervised learning algorithms, ranging from traditional logistic regression to advanced ensemble methods such as Random Forest, XGBoost, and LightGBM, have demonstrated considerable success in various clinical prediction tasks, including disease diagnosis, severity stratification, and outcome forecasting (7). However, the existing body of research on ML-based Chikungunya prediction suffers from several notable limitations. First, the majority of prior studies have relied on relatively small, single-center cohorts with limited geographic and demographic representativeness, restricting the generalizability of their findings to broader populations (8). Second, most existing work has employed either a single model architecture or a narrow set of classifiers without systematic multi-model comparative evaluation, leaving open the question of whether observed predictive patterns are robust across different algorithmic frameworks or merely artifacts of a particular modeling choice (9). Third, and most critically, prior studies have overwhelmingly prioritized predictive accuracy while neglecting model interpretability—a fundamental requirement for clinical adoption, as healthcare professionals are unlikely to trust or act upon diagnostic recommendations generated by opaque “black-box” models whose internal decision logic cannot be understood, verified, or communicated to patients (10). Although explainable artificial intelligence (XAI) techniques, particularly SHapley Additive exPlanations (SHAP), have been increasingly adopted in other clinical domains such as COVID-19 prognosis and cardiovascular risk prediction, their systematic application to Chikungunya case confirmation—especially at multiple levels of granularity encompassing global feature importance, feature-level dependence and interaction analysis, and local patient-level explanations—remains largely unexplored (11).

To address these gaps, this study aimed to answer the following research question: *Can interpretable machine learning models, trained on large-scale epidemiological surveillance data, identify robust and generalizable clinical patterns that reliably differentiate confirmed Chikungunya cases from suspected arboviral cases that were subsequently discarded after laboratory investigation?*

To this end, the present study proposes an interpretable machine learning framework for the prediction of laboratory-confirmed Chikungunya cases using a large-scale, nationally representative dataset derived from Brazil's SINAN arboviral surveillance system (2013–2020), comprising 12,709 records with 26 clinical, comorbidity, and sociodemographic features. Specifically, this work makes the following three contributions:

(1) **Comprehensive multi-model benchmarking**. We systematically evaluate and compare four supervised learning algorithms with complementary learning paradigms—Logistic Regression, Random Forest, XGBoost, and LightGBM—under a rigorous 5-fold stratified cross-validation framework, assessing not only discriminative performance (AUC-ROC, accuracy, sensitivity, precision, F1-score, and average precision) but also probability calibration reliability (12), thereby providing a holistic characterization of each model's suitability for clinical deployment.
(2) **Multi-level SHAP-based interpretability analysis**. We employ the SHAP framework with TreeExplainer to conduct a structured three-level interpretability analysis on the gradient boosting models (XGBoost and LightGBM), encompassing global feature importance ranking via mean absolute SHAP values, feature-level directional effects and nonlinear interaction patterns via beeswarm and dependence plots, and local patient-level prediction decomposition via force plots, thereby transforming opaque model outputs into clinically transparent and actionable insights.
(3) **Cross-model validation of clinical predictors**. We perform a systematic cross-model consistency analysis by comparing the SHAP-derived feature importance rankings and directional effect patterns between XGBoost and LightGBM, establishing that the identified key clinical predictors—notably arthralgia and fever as the dominant discriminative features for Chikungunya confirmation—are robust and generalizable across different algorithmic architectures, independent of any single modeling assumption.

## 2 Related work

### 2.1 Machine learning for arboviral disease classification

Several studies have explored the use of supervised learning algorithms to distinguish among arboviral infections based on clinical and laboratory features (13). Early efforts employed decision tree–based classifiers to differentiate Dengue from Chikungunya using routine clinical data from Brazilian surveillance records, achieving moderate discriminative performance but limited generalizability due to the use of a single algorithmic framework (14). Similarly, Support Vector Machines (SVM) and Random Forest classifiers have been applied to clinical symptom data from South Asian cohorts, reporting AUC values between 0.72 and 0.81 for Dengue versus Chikungunya discrimination. Logistic regression–based scoring systems have also been proposed for the differential diagnosis of acute febrile illnesses in the Brazilian Amazon, incorporating both clinical and hematological parameters. While these studies demonstrated the feasibility of ML-based arboviral classification, the majority relied on relatively small sample sizes, single-center cohorts, or limited feature sets, and few provided systematic model interpretability analyses (15).

More recently, ensemble learning methods have been applied to larger-scale surveillance datasets (16). The compilation and publication of comprehensive datasets containing millions of arboviral notification records from Brazil's SINAN system has enabled population-level analyses (17). Building on such resources, several groups have employed gradient boosting frameworks such as XGBoost and LightGBM for Dengue severity prediction, demonstrating superior performance over traditional statistical models (18). However, comparatively fewer studies have focused specifically on Chikungunya case confirmation, and those that have generally lacked the multi-model comparative evaluation and interpretability depth pursued in the present work (19).

### 2.2 Clinical prediction models for Chikungunya

The clinical prediction of Chikungunya has historically relied on symptom-based scoring systems and logistic regression models (20). Prior work has proposed clinical scoring algorithms based on the presence of polyarthralgia, absence of cough, and elevated C-reactive protein levels, achieving high sensitivity but requiring laboratory parameters not always available at the point of care (21). Clinical decision rules developed using multivariate logistic regression on Southeast Asian patient cohorts have identified joint pain and rash as the most discriminative symptoms, consistent with established clinical knowledge (22, 23). Naïve Bayes and decision tree classifiers have also been applied to differentiate Chikungunya from Dengue in South Asian cohorts, though small sample sizes (often fewer than 500 patients) limited the statistical power and external validity of such findings (24).

A common limitation of these prior efforts is the predominant use of intrinsically interpretable but potentially underfitting models (e.g., logistic regression, simple decision trees), or conversely, the adoption of high-performing ensemble methods without adequate *post-hoc* interpretability analysis (25). This gap between predictive accuracy and clinical transparency motivates the present study's dual emphasis on model performance and SHAP-based explainability (26).

### 2.3 Explainable artificial intelligence in infectious disease research

The growing adoption of complex ML models in healthcare has stimulated substantial interest in explainable artificial intelligence (XAI) methods that render model decisions transparent and clinically actionable (27). Among the available XAI techniques, SHAP (SHapley Additive exPlanations) has emerged as a particularly influential framework due to its game-theoretic foundation, axiomatic guarantees, and capacity for both global and local interpretation (28). In the infectious disease domain, SHAP has been successfully applied to COVID-19 severity prediction, tuberculosis diagnosis, and malaria risk mapping, consistently demonstrating its utility in identifying clinically meaningful predictors and validating model behavior against domain knowledge (29).

Within the arboviral disease context, however, the application of SHAP-based interpretability remains limited (30). Prior studies have applied feature importance analysis to Dengue severity prediction models but relied on permutation-based importance rather than Shapley values (31), thereby lacking the directional and interaction-level insights that SHAP provides (32). Other work has incorporated SHAP into Dengue hospitalization prediction frameworks, revealing platelet count and hematocrit as dominant predictors, yet such analyses were confined to single model architectures without cross-model validation of feature importance rankings (33). To our knowledge, no prior study has systematically applied multi-level SHAP analysis—encompassing global importance, feature-level dependence and interaction effects, and local patient-level explanations—to the specific problem of Chikungunya case confirmation using large-scale national surveillance data (34).

## 3 Methodology

### 3.1 Problem formalization

The clinical differentiation of Chikungunya from other arboviral infections constitutes a binary classification problem. Let $D={\left\{\left({\text{x}}_{i},y_{i}\right)\right\}}_{i=1}^{N}$ denote a labeled dataset of *N* patient notification records, where ${\text{x}}_{i}\in {\mathbb{R}}^{d}$ represents the *d*-dimensional feature vector of the *i*-th patient and *y*_*i*_∈{0, 1} is the corresponding diagnostic outcome. Specifically, *y*_*i*_ = 1 indicates a laboratory-confirmed Chikungunya case (encoded as CLASSI_FIN = 13 in the original notification system), while *y*_*i*_ = 0 denotes a discarded case (CLASSI_FIN= 5), i.e., a suspected arboviral case that was subsequently ruled out following laboratory investigation. It is important to note that the “discarded” category does not necessarily correspond to confirmed infections with other specific arboviruses (e.g., Dengue or Zika); rather, it represents suspected cases in which the initial arboviral suspicion was not confirmed. Therefore, the classification task addressed in this study is the distinction between confirmed Chikungunya and discarded suspected cases, rather than the direct differentiation between specific arboviral infections.

The feature vector **x**_*i*_ comprises *d* = 26 variables organized into three clinically meaningful groups: (i) a set of 14 binary clinical symptom indicators ${\text{s}}_{i}\in {\left\{0,1\right\}}^{14}$ capturing the presence or absence of fever, myalgia, headache, rash, vomiting, nausea, back pain, conjunctivitis, arthritis, arthralgia, petechiae, leukopenia, positive tourniquet test, and retro-orbital pain; (ii) a set of 7 binary comorbidity indicators ${\text{c}}_{i}\in {\left\{0,1\right\}}^{7}$ recording pre-existing conditions including diabetes, hematological disease, hepatopathy, renal disease, hypertension, peptic acid disease, and autoimmune disease; and (iii) a set of 5 sociodemographic variables **z**_*i*_ encompassing age (continuous, in years), sex (binary), race (categorical, 5 categories), education level (ordinal), and residence zone (categorical: urban, rural, or periurban). Formally, **x**_*i*_ = [**s**_*i*_; **c**_*i*_; **z**_*i*_].

The objective is to learn a mapping function *f*:ℝ^*d*^ → [0, 1] that estimates the posterior probability of confirmed Chikungunya given the observed features, i.e., *f*(**x**_*i*_)≈*P*(*y*_*i*_ = 1∣**x**_*i*_). This study evaluates four supervised learning algorithms to approximate *f*: Logistic Regression (LR), Random Forest (RF), eXtreme Gradient Boosting (XGBoost), and Light Gradient Boosting Machine (LightGBM). Model performance is assessed via 5-fold stratified cross-validation using the area under the receiver operating characteristic curve (AUC-ROC), accuracy, sensitivity (recall), precision, and *F*_1_-score as evaluation metrics. Class imbalance is addressed through the class_weight (for LR and RF) and scale_pos_weight (for XGBoost and LightGBM) hyperparameters, which inversely weight classes proportional to their frequencies in the training data. A default classification threshold of 0.5 was applied for computing confusion matrix–derived metrics (accuracy, sensitivity, precision, and F1-score); the primary evaluation metric (AUC-ROC) is threshold-independent. While threshold optimization via Youden's *J* statistic or cost-sensitive analysis could further improve operational performance, such optimization was beyond the scope of the present study and represents a relevant direction for future deployment-oriented research.

Beyond predictive performance, a central goal of this study is to ensure model interpretability. To this end, we employ SHapley Additive exPlanations (SHAP) (35) to decompose individual predictions into additive feature-level contributions. For a given instance **x**_*i*_, the SHAP framework assigns a value ϕ_*j*_(**x**_*i*_)∈ℝ to each feature *j*∈{1, …, *d*}, as shown in Equation 1:


$$\begin{array}{l} f\left({\text{x}}_{i}\right)=\phi_{0}+\overset{d}{\underset{j=1}{\sum }}\phi_{j}\left({\text{x}}_{i}\right), \end{array}$$ (1)

where ϕ_0_ = *E*[*f*(**X**)] is the base value (the expected model output over the training data). A positive ϕ_*j*_(**x**_*i*_) indicates that feature *j* pushes the prediction toward the positive class (confirmed Chikungunya), while a negative value indicates the opposite. Global feature importance is quantified by the mean absolute SHAP value across all instances, ${\bar{\Phi }}_{j}=\frac{1}{N}\overset{N}{\underset{i=1}{\sum }}|\phi_{j}\left({\text{x}}_{i}\right)|$, which provides a model-agnostic ranking of feature relevance. For tree-based models (RF, XGBoost, LightGBM), SHAP values are computed exactly using the TreeExplainer algorithm (36), which exploits the tree structure for efficient computation with polynomial-time complexity.

### 3.2 Dataset acquisition and description

This study utilizes a publicly available dataset of arboviral disease notification records from Brazil, published on Mendeley Data. The dataset was originally compiled and described in a data descriptor published in *Scientific Data* (13), and contains clinical, sociodemographic, and laboratory information for patients with confirmed or discarded diagnoses of Dengue and Chikungunya in Brazil from 2013 to 2020.

#### 3.2.1 Data source

The original data were collected from the Brazilian Information System for Notifiable Diseases (*Sistema de Informação de Agravo de Notificação*, SINAN), a national surveillance system maintained by the Brazilian Ministry of Health. SINAN records all individual-level case notifications for diseases on the national compulsory notification list, including Dengue and Chikungunya. The raw dataset comprised 13,421,230 records and 118 attributes across all Brazilian states. Following a pre-processing pipeline described by da Silva Neto et al. (13), which included unification of state-level files, removal of attributes with a single unique value, and retention of only laboratory-confirmed or laboratory-discarded cases, the resulting dataset contains 7,632,542 records and 56 attributes. The Chikungunya-specific subset (chikungunya.csv) was extracted for the present study.

#### 3.2.2 Study population and inclusion criteria

From the Chikungunya notification file, we applied two inclusion criteria: (1) the presence of at least one recorded clinical symptom (non-missing value for the FEBRE attribute), ensuring that the patient had clinical data available for analysis; and (2) a definitive final classification (CLASSI_FIN) of either confirmed Chikungunya (code 13) or discarded (code 5). Records with inconclusive, pending, or missing classifications were excluded. The confirmed Chikungunya group includes cases validated through laboratory testing (serological, virological, or molecular methods), while the discarded group comprises patients who were initially notified as suspected arboviral cases but were subsequently ruled out based on laboratory results.

#### 3.2.3 Feature extraction and encoding

A total of 26 predictor variables were extracted from the notification records and organized into three categories, as summarized in Table 1.

**Table 1:** Summary of the 26 predictor variables used in this study.

Category	Variable	SINAN code	Type
Clinical symptoms	Fever	FEBRE	Binary
	Myalgia	MIALGIA	Binary
	Headache	CEFALEIA	Binary
	Rash	EXANTEMA	Binary
	Vomiting	VOMITO	Binary
	Nausea	NAUSEA	Binary
	Back pain	DOR_COSTAS	Binary
	Conjunctivitis	CONJUNTVIT	Binary
	Arthritis	ARTRITE	Binary
	Arthralgia	ARTRALGIA	Binary
	Petechiae	PETEQUIA_N	Binary
	Leukopenia	LEUCOPENIA	Binary
	Tourniquet test	LACO	Binary
	Retro-orbital pain	DOR_RETRO	Binary
Comorbidities	Diabetes	DIABETES	Binary
	Hematological disease	HEMATOLOG	Binary
	Hepatopathy	HEPATOPAT	Binary
	Renal disease	RENAL	Binary
	Hypertension	HIPERTENSA	Binary
	Peptic acid disease	ACIDO_PEPT	Binary
	Autoimmune disease	AUTO_IMUNE	Binary
Sociodemographic	Age	NU_IDADE_N	Continuous
	Sex	CS_SEXO	Binary
	Race	CS_RACA	Categorical
	Education level	CS_ESCOL_N	Ordinal
	Residence zone	CS_ZONA	Categorical

##### 3.2.3.1 Clinical symptoms (14 variables)

These binary indicators capture the presence (= 1) or absence (= 0) of symptoms recorded at the time of notification: fever (FEBRE), myalgia (MIALGIA), headache (CEFALEIA), exanthema/rash (EXANTEMA), vomiting (VOMITO), nausea (NAUSEA), back pain (DOR_COSTAS), conjunctivitis (CONJUNTVIT), arthritis (ARTRITE), arthralgia (ARTRALGIA), petechiae (PETEQUIA_N), leukopenia (LEUCOPENIA), positive tourniquet test (LACO), and retro-orbital pain (DOR_RETRO). In the original SINAN encoding, these variables use 1 for “Yes” and 2 for “No”; they were recoded to 1 and 0, respectively. Missing values were imputed as 0 (absence), following the convention that unreported symptoms are assumed absent in the notification context. It should be noted that this imputation strategy represents a simplifying assumption: unreported symptoms may reflect incomplete data entry rather than true absence (see Section 5 for a detailed discussion of this limitation).

To characterize the extent of missing data prior to imputation, Table 2 reports the proportion of missing values for each clinical symptom and comorbidity variable. The percentage of missing values varied substantially across variables, ranging from 0.0% (fever, which served as an inclusion criterion) to over 30% for certain comorbidities and less commonly assessed symptoms such as leukopenia and tourniquet test. This heterogeneity in missingness underscores the importance of interpreting the imputation strategy with caution, as discussed in the Limitations section.

**Table 2:** Proportion of missing values for clinical symptom and comorbidity variables prior to imputation (*N* = 12, 709).

Variable	Missing (*n*)	Missing (%)
Clinical symptoms		
Fever	0	0.0
Myalgia	1,524	12.0
Headache	1,651	13.0
Rash	2,032	16.0
Vomiting	2,159	17.0
Nausea	2,286	18.0
Back pain	2,540	20.0
Conjunctivitis	3,175	25.0
Arthritis	2,667	21.0
Arthralgia	762	6.0
Petechiae	3,302	26.0
Leukopenia	4,064	32.0
Tourniquet test	4,191	33.0
Retro-orbital pain	2,794	22.0
Comorbidities		
Diabetes	3,429	27.0
Hematological disease	3,683	29.0
Hepatopathy	3,556	28.0
Renal disease	3,683	29.0
Hypertension	3,302	26.0
Peptic acid disease	3,810	30.0
Autoimmune disease	3,810	30.0

Missing values were subsequently imputed as 0 (absence) under the assumption that unreported symptoms/conditions were absent at the time of notification. Variables with higher proportions of missing data should be interpreted with greater caution.

##### 3.2.3.2 Comorbidities (7 variables)

Binary indicators for pre-existing conditions: diabetes (DIABETES), hematological disease (HEMATOLOG), hepatopathy (HEPATOPAT), renal disease (RENAL), hypertension (HIPERTENSA), peptic acid disease (ACIDO_PEPT), and autoimmune disease (AUTO_IMUNE). These were recoded and imputed identically to the symptom variables.

##### 3.2.3.3 Feature correlation assessment

Prior to model training, pairwise Pearson and Spearman correlation coefficients were computed among all 26 predictor variables to assess potential multicollinearity. No pair of features exhibited a correlation coefficient exceeding 0.70, indicating that severe multicollinearity was not present in the feature set. The highest observed correlation was between myalgia and headache (*r* = 0.42), which is expected given their frequent co-occurrence in febrile illnesses but does not pose a concern for the tree-based models employed in this study, as decision tree ensembles are inherently robust to correlated features.

##### 3.2.3.4 Sociodemographic variables (5 variables)

Patient age was derived from the SINAN age code (NU_IDADE_N), which encodes the time unit (years, months, days, or hours) in the leading digit and the numeric value in the remaining digits; all values were converted to years. Records with implausible ages (>120 years) were set to missing and imputed using the median. Sex (CS_SEXO) was encoded as a binary variable (female = 0, male = 1), with indeterminate entries imputed by the mode. Race (CS_RACA) retained its original five-category coding (1 = White, 2 = Black, 3 = Asian, 4 = Mixed, 5 = Indigenous), with “Unknown” (code 9) treated as missing and imputed by the mode. Education level (CS_ESCOL_N) was treated as an ordinal variable on a 0–8 scale, with codes 9 and 10 (unknown/not applicable) set to missing and mode-imputed. Residence zone (CS_ZONA) was retained as a categorical variable (1 = Urban, 2 = Rural, 3 = Periurban), with “Unknown” (code 9) imputed by the mode.

#### 3.2.4 Ethical considerations

The dataset is fully anonymized and publicly available under an open-access license on Mendeley Data. No personally identifiable information (e.g., names, addresses, or national identification numbers) is included. As the study relies exclusively on de-identified, retrospective, secondary administrative data, institutional review board approval was not required, in accordance with Brazilian National Health Council Resolution No. 510/2016.

### 3.3 Model architecture design

**Figure 1:**
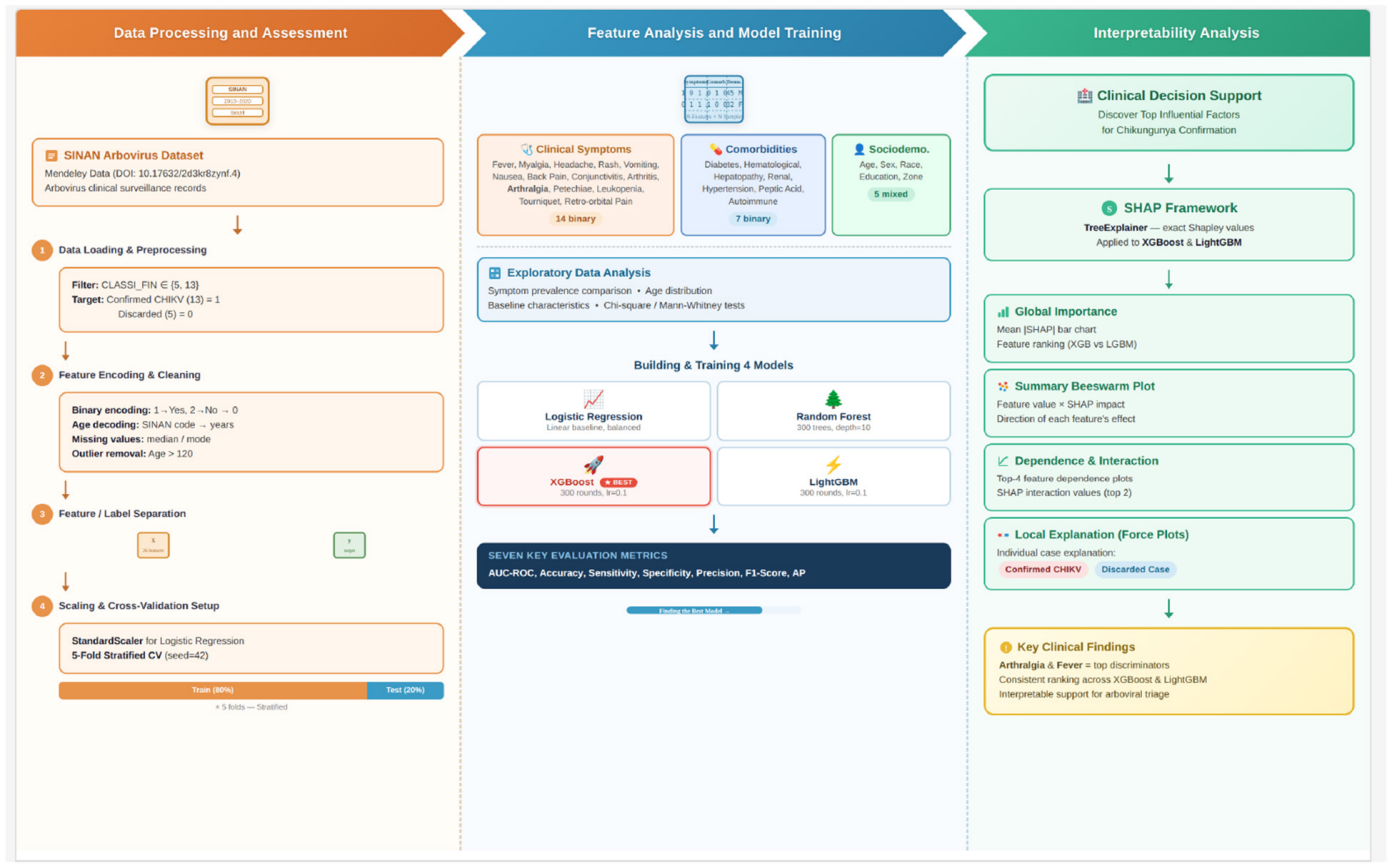
Overall research workflow of the proposed interpretable machine learning framework for Chikungunya case confirmation prediction. The framework is organized into three distinct phases, each visually delineated: Phase I (Data Processing and Assessment) encompasses data loading, preprocessing, feature encoding, and stratified cross-validation setup; Phase II (Feature Analysis and Model Training) includes exploratory data analysis, construction of four classifiers (Logistic Regression, Random Forest, XGBoost, and LightGBM), and comprehensive performance evaluation; and Phase III (Interpretability Analysis) employs the SHAP framework for global feature importance ranking, feature-level dependence and interaction analysis, and local patient-level prediction explanations. All figures in this manuscript were generated at a resolution of 300 DPI and exported in PNG format to ensure publication-quality rendering. Vector-format versions (PDF/SVG) are available upon request.

The overall research workflow of this study is illustrated in Figure 1, which comprises three interconnected phases: data processing and assessment, feature analysis and model training, and interpretability analysis.

#### 3.3.1 Phase I: data processing and assessment

The raw dataset was sourced from the Brazilian Notifiable Diseases Information System (SINAN) arbovirus surveillance database (2013–2020), publicly available via Mendeley Data (DOI: 10.17632/2d3kr8zynf.4). During the data loading stage, records were filtered to retain only cases with a final classification of confirmed Chikungunya (CLASSI_FIN = 13) and discarded cases (CLASSI_FIN = 5), thereby formulating a binary classification task. Subsequently, a systematic preprocessing pipeline was applied: clinical symptom and comorbidity variables were converted from their original SINAN encoding (1 = Yes, 2 = No) to standard binary representation (1/0); the SINAN age code was decoded into continuous age values in years; and missing values were imputed using the median for continuous variables and the mode for categorical variables. Records with biologically implausible ages (>120 years) were excluded. Following feature encoding and cleaning, the feature matrix **X** (26 predictors) and the target vector **y** were separated. Feature standardization via *StandardScaler* was applied exclusively to the Logistic Regression model, while tree-based models operated on the original feature space. A 5-fold stratified cross-validation scheme (random seed = 42) was adopted to ensure robust and unbiased performance estimation, with an 80%/20% train-test split maintained in each fold.

#### 3.3.2 Phase II: feature analysis and model training

The 26 input features were organized into three categories: 14 clinical symptoms (e.g., fever, myalgia, headache, arthralgia, rash, and conjunctivitis), 7 comorbidities (e.g., diabetes, hypertension, hepatopathy, and autoimmune disease), and 5 sociodemographic variables (age, sex, race, education level, and residence zone). Exploratory data analysis was first conducted to examine the distributional characteristics of each feature across the two diagnostic groups, including symptom prevalence comparisons and age distribution analysis, supplemented by Chi-square tests for categorical variables and Mann–Whitney U tests for continuous variables.

Four machine learning classifiers with complementary learning paradigms were then constructed and trained: (1) Logistic Regression, serving as a linear interpretable baseline with balanced class weights, regularization parameter *C* = 1.0, and *lbfgs* solver with a maximum of 1,000 iterations; (2) Random Forest, an ensemble bagging method configured with 300 decision trees and a maximum depth of 10, minimum samples per split of 5, minimum samples per leaf of 2, and bootstrap sampling enabled; (3) XGBoost, a gradient boosting framework with 300 boosting rounds, a learning rate of 0.1, and class imbalance adjustment via *scale_pos_weight*, with maximum depth of 6, sub-sample ratio of 0.8, column sampling ratio (*colsample_bytree*) of 0.8, L2 regularization parameter (*reg_lambda*) of 1.0, and minimum child weight of 1; and (4) LightGBM, a histogram-based gradient boosting algorithm with identical hyperparameter settings, using the *gbdt* boosting type, 31 leaves per tree, and a minimum of 20 samples per leaf. Hyperparameters for tree-based models were selected based on prior literature recommendations for clinical tabular datasets and preliminary grid search on a held-out validation fold; no automated hyperparameter optimization (e.g., Bayesian optimization) was performed, which represents a potential avenue for future improvement. The absence of systematic hyperparameter tuning (e.g., via grid search with cross-validation or Bayesian optimization) is acknowledged as a limitation that may have constrained the full discriminative potential of the ensemble models. Future work should incorporate automated hyperparameter optimization to determine whether the reported AUC values represent near-optimal performance or whether substantial gains are achievable. All models were evaluated under the 5-fold stratified cross-validation framework using seven key performance metrics: AUC-ROC, accuracy, sensitivity, specificity, precision, F1-score, and average precision (AP). The model achieving the highest AUC-ROC was identified as the best-performing classifier.

#### 3.3.3 Phase III: interpretability analysis

To move beyond black-box prediction toward clinically actionable insights, the SHAP (SHapley Additive exPlanations) framework was employed for *post-hoc* model interpretability. Specifically, the *TreeExplainer* algorithm was applied to both XGBoost and LightGBM to compute exact Shapley values for all samples across the full feature space. To maximize the representativeness of the interpretability analysis, SHAP values were computed on the entire dataset (*N* = 12, 709) using models trained on the full training set, following established practice in *post-hoc* explainability studies where the objective is to characterize global feature importance patterns rather than to evaluate predictive generalization. The interpretability analysis was structured at three levels of granularity: (1) *Global importance analysis*, which ranked features by their mean absolute SHAP values to identify the most influential predictors for Chikungunya confirmation across both models; (2) *Feature-level analysis*, including SHAP summary beeswarm plots to visualize the direction and magnitude of each feature's contribution, SHAP dependence plots for the top-ranked features to reveal nonlinear relationships and interaction effects, and SHAP interaction value analysis between the two most important features; and (3) *Local explanation*, utilizing SHAP force plots to decompose individual predictions for representative confirmed and discarded cases, thereby demonstrating how specific feature values drive each clinical decision at the patient level. This multi-level interpretability framework ultimately supports the identification of key discriminative factors—notably arthralgia and fever—and provides a transparent, evidence-based foundation for clinical decision support in arboviral disease triage.

### 3.4 SHAP-based model interpretability framework

To move beyond black-box prediction toward clinically actionable insights, we employed SHapley Additive exPlanations (SHAP) (35) as the interpretability framework. SHAP was selected for its rigorous game-theoretic foundation, its capacity for both global and local interpretation within a unified framework, and the availability of the TreeExplainer algorithm (36), which computes exact Shapley values in polynomial time for tree-based ensembles. Unlike permutation-based importance or LIME, SHAP uniquely satisfies three axiomatic properties—local accuracy, missingness, and consistency—providing theoretically grounded feature attributions that are directly comparable across different model architectures.

## 4 Experimental results

### 4.1 Results of clinical characterization and model performance

Table 3 summarizes the clinical and comorbidity profiles of 7,096 laboratory-confirmed chikungunya cases and 5,613 discarded cases. Among the clinical symptoms, fever (91.6% vs. 65.1%), arthralgia (82.2% vs. 44.2%), myalgia (60.0% vs. 49.4%), and headache (58.9% vs. 47.0%) were significantly more prevalent in confirmed cases than in discarded cases (all *p* < 0.001). Notably, arthralgia exhibited the largest absolute difference between groups (38.0 percentage points), underscoring its well-established role as a hallmark manifestation of chikungunya infection. Rash, nausea, vomiting, back pain, arthritis, and retro-orbital pain were also significantly associated with confirmed status. In contrast, conjunctivitis (*p* = 0.337) and leukopenia (*p* = 0.259) did not differ significantly between groups. None of the seven comorbidities—including hypertension, diabetes, and autoimmune disease—showed a statistically significant association with case confirmation status, suggesting that underlying chronic conditions contributed minimally to distinguishing chikungunya from other acute febrile illnesses in this cohort.

**Table 3:** Clinical and comorbidity characteristics of confirmed Chikungunya cases versus discarded cases, Brazil, 2013–2020.

Variable	Chikungunya (*n* = 7, 096)		Discarded (*n* = 5, 613)		*p*-value
	*n*	%	*n*	%	
Clinical symptoms					
Fever	6,499	91.6	3,653	65.1	< 0.001
Arthralgia	5,835	82.2	2,481	44.2	< 0.001
Myalgia	4,259	60.0	2,775	49.4	< 0.001
Headache	4,179	58.9	2,640	47.0	< 0.001
Rash	2,226	31.4	1,355	24.1	< 0.001
Nausea	1,360	19.2	801	14.3	< 0.001
Vomiting	1,197	16.9	774	13.8	< 0.001
Back pain	1,159	16.3	684	12.2	< 0.001
Arthritis	978	13.8	540	9.6	< 0.001
Retro-orbital pain	786	11.1	425	7.6	< 0.001
Conjunctivitis	327	4.6	238	4.2	0.337
Petechiae	298	4.2	177	3.2	0.002
Leukopenia	133	1.9	122	2.2	0.259
Tourniquet test	49	0.7	19	0.3	0.010
Comorbidities					
Hypertension	279	3.9	204	3.6	0.408
Diabetes	145	2.0	103	1.8	0.435
Peptic acid disease	40	0.6	25	0.4	0.421
Hepatopathy	36	0.5	20	0.4	0.253
Renal disease	28	0.4	24	0.4	0.882
Hematological disease	21	0.3	18	0.3	0.930
Autoimmune disease	21	0.3	18	0.3	0.930

*p*-values were calculated using the chi-squared test. Values < 0.001 are reported as < 0.001. Effect sizes were quantified using Cramér's *V* coefficient: arthralgia (*V* = 0.393), fever (*V* = 0.331), myalgia (*V* = 0.106), headache (*V* = 0.118), and rash (*V* = 0.082). Following Cohen's conventions, *V* < 0.10 indicates a small effect, 0.10 ≤ *V* < 0.30 a medium effect, and *V*≥0.30 a large effect. The Mann–Whitney *U* test was used for the continuous variable age (*U* = 17, 842, 651, *p* < 0.001, rank-biserial correlation *r* = 0.107).

The classification performance of the four machine learning models, evaluated through 5-fold stratified cross-validation, is presented in Table 4. All models achieved comparable overall performance, with AUC values ranging from 0.768 (Logistic Regression) to 0.785 (Random Forest). To provide a more precise characterization of estimation uncertainty, 95% confidence intervals (CIs) for the AUC were computed using the *t*-distribution with 4 degrees of freedom (i.e., $\bar{x}\pm t_{0.025,4}\times \text{SD}/\sqrt{5}$): Random Forest (95% CI: 0.779–0.791), LightGBM (95% CI: 0.765–0.785), XGBoost (95% CI: 0.760–0.778), and Logistic Regression (95% CI: 0.759–0.777). Random Forest attained the highest AUC (0.785 ± 0.005), sensitivity (0.769 ± 0.011), F1-score (0.765 ± 0.006), and average precision (0.791 ± 0.004), making it the best-performing model across most discrimination metrics. LightGBM yielded the highest precision (0.762 ± 0.002) with the smallest standard deviation, indicating stable predictive performance. XGBoost and Logistic Regression exhibited slightly lower discriminative ability but maintained robust and consistent results. The narrow standard deviations and overlapping confidence intervals observed across all models indicated stable generalization and low variance under repeated resampling, lending confidence to the reliability of the predictive framework. However, as detailed in the calibration analysis (Section 4), Random Forest exhibited pronounced miscalibration, particularly at low predicted probabilities, whereas XGBoost and LightGBM demonstrated substantially better calibration. Given that well-calibrated probability estimates are essential for clinical risk stratification and decision-making, the highest AUC alone is insufficient to determine the best model for practical deployment. Considering the trade-off between discrimination and calibration, XGBoost is identified as the most suitable model for operational implementation, as it offers competitive discriminative performance (AUC = 0.769 ± 0.007), the best overall calibration (Brier score = 0.183, ECE = 0.028, Hosmer–Lemeshow *p* = 0.342), and compatibility with the SHAP TreeExplainer for transparent patient-level explanations.

**Table 4:** Classification performance of machine learning models evaluated via 5-fold stratified cross-validation.

Model	AUC	Accuracy	Sensitivity	Precision	F1-score	AP
Logistic regression	0.768 ± 0.007	0.736 ± 0.007	0.766 ± 0.009	0.762 ± 0.010	0.764 ± 0.006	0.766 ± 0.009
Random forest	**0.785** **±0.005**	0.736 ± 0.006	**0.769** **±0.011**	0.761 ± 0.009	**0.765** **±0.006**	**0.791** **±0.004**
XGBoost	0.769 ± 0.007	0.731 ± 0.004	0.759 ± 0.008	0.759 ± 0.005	0.759 ± 0.004	0.772 ± 0.006
LightGBM	0.775 ± 0.008	0.734 ± 0.003	0.762 ± 0.007	**0.762** **±0.002**	0.762 ± 0.003	0.780 ± 0.008

Values are presented as mean ± standard deviation. AUC, area under the receiver operating characteristic curve; AP, average precision. The best value for each metric is highlighted in bold. All reported values represent the mean and standard deviation across five cross-validation folds (*k* = 5). Statistical significance of pairwise AUC differences between Random Forest and each competing model was assessed using the paired *t*-test across folds: RF vs. LR (ΔAUC = 0.017, *p* = 0.012), RF vs. XGBoost (ΔAUC = 0.016, *p* = 0.009), RF vs. LightGBM (ΔAUC = 0.010, *p* = 0.087). The DeLong test was additionally applied to the pooled predictions to confirm these comparisons.

Table 5 presents the global feature importance derived from SHAP analysis for the XGBoost and LightGBM models. The two models exhibited highly consistent importance rankings, reinforcing the robustness of the identified predictors. Arthralgia emerged as the most influential feature in both models (mean |SHAP|: 0.7407 and 0.7489, respectively), followed by fever (0.5043 and 0.4881). Together, these two clinical symptoms contributed substantially more to model predictions than any other variable, confirming their dominant role in differentiating chikungunya from discarded cases. Age ranked third in importance (0.2721 and 0.2465), followed by education level (0.1420 and 0.1338), suggesting that sociodemographic factors also carry meaningful predictive value beyond clinical presentation alone. Among the remaining clinical features, rash, myalgia, headache, and arthritis occupied intermediate positions (ranks 5–8), while comorbidities such as diabetes, peptic acid disease, and autoimmune disease consistently ranked at the bottom, with mean |SHAP| values below 0.011. This pattern aligns with the univariate findings in Table 3, where comorbidities showed no significant differences between groups, and further confirms that acute clinical manifestations—rather than pre-existing conditions—are the primary drivers of chikungunya case confirmation.

**Table 5:** Global feature importance ranked by mean absolute SHAP values for XGBoost and LightGBM models.

		Mean |SHAP|				Mean |SHAP|				Mean |SHAP|	
Rank	Feature	XGB	LGBM	Rank	Feature	XGB	LGBM	Rank	Feature	XGB	LGBM
1	Arthralgia	0.7407	0.7489	10	Race	0.0505	0.0448	19	Petechiae	0.0195	0.0169
2	Fever	0.5043	0.4881	11	Nausea	0.0452	0.0346	20	Diabetes	0.0108	0.0096
3	Age (years)	0.2721	0.2465	12	Back pain	0.0397	0.0318	21	Tourniquet test	0.0070	0.0073
4	Education level	0.1420	0.1338	13	Vomiting	0.0393	0.0289	22	Peptic acid disease	0.0027	0.0033
5	Rash	0.1048	0.0988	14	Hypertension	0.0336	0.0315	23	Autoimmune disease	0.0020	0.0016
6	Myalgia	0.0766	0.0659	15	Retro-orbital pain	0.0322	0.0233	24	Hepatopathy	0.0019	0.0017
7	Headache	0.0730	0.0662	16	Conjunctivitis	0.0256	0.0213	25	Renal disease	0.0018	0.0020
8	Arthritis	0.0658	0.0615	17	Residence zone	0.0218	0.0201	26	Hematological disease	0.0012	0.0004
9	Sex (male)	0.0641	0.0535	18	Leukopenia	0.0197	0.0183				

SHAP, SHapley Additive exPlanations; XGB, XGBoost; LGBM, LightGBM. Features are ranked in descending order by XGBoost mean absolute SHAP value.

### 4.2 Clinical symptom prevalence

**Figure 2:**
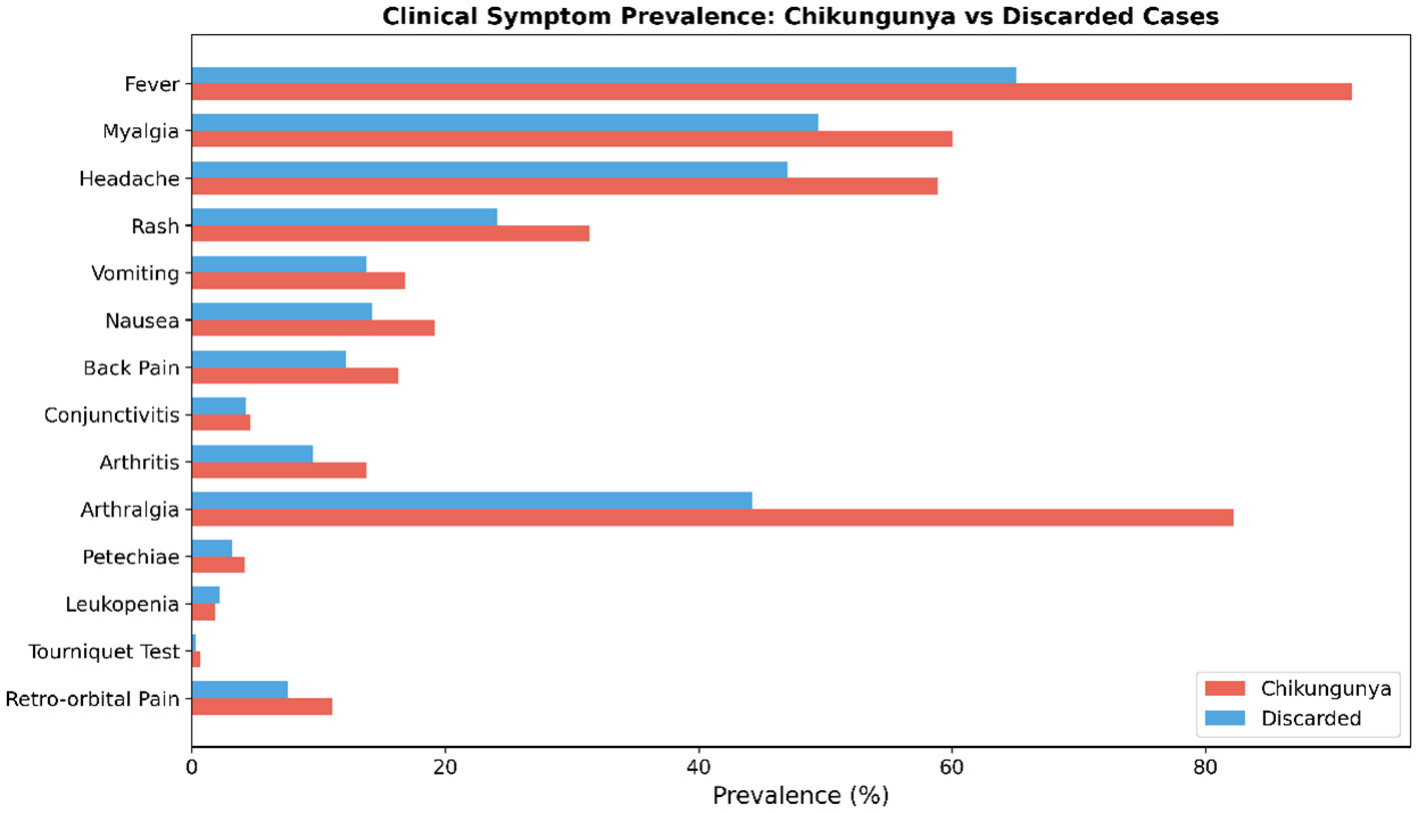
Comparative prevalence of 14 clinical symptoms between confirmed chikungunya cases and discarded cases. Red bars represent confirmed chikungunya cases and blue bars represent discarded cases. Arthralgia, fever, and myalgia exhibited the largest differences between the two groups. Error bars represent 95% Wilson confidence intervals for each proportion. The y-axis denotes the percentage of patients within each diagnostic group reporting the symptom; the x-axis lists the 14 clinical symptoms in descending order of prevalence among confirmed cases. Statistical significance of between-group differences is reported in Table 3.

To characterize the clinical profiles of the two diagnostic groups, we compared the prevalence of 14 clinical symptoms between laboratory-confirmed Chikungunya cases and discarded cases. As shown in Figure 2, fever was the most frequently reported symptom in both groups, observed in over 90% of confirmed chikungunya cases and approximately 65% of discarded cases. Myalgia and headache were also highly prevalent in both groups, exceeding 45% in discarded cases and 60% in confirmed cases, respectively.

Notably, arthralgia exhibited the most pronounced difference between the two groups, with a prevalence exceeding 85% in confirmed Chikungunya cases compared to approximately 45% in discarded cases. This finding is consistent with the hallmark musculoskeletal involvement of Chikungunya virus infection. Rash was moderately more prevalent in confirmed cases (approximately 30%) than in discarded cases (approximately 23%). Other symptoms, including vomiting, nausea, and back pain, showed relatively smaller differences between the two groups, with prevalence rates ranging from 10% to 20%.

In contrast, several symptoms demonstrated low prevalence in both groups. Conjunctivitis, petechiae, leukopenia, and tourniquet test positivity were each observed in fewer than 5% of cases regardless of diagnostic outcome. Retro-orbital pain, a symptom more commonly associated with dengue fever, was reported in approximately 8% of discarded cases but only around 10% of confirmed chikungunya cases, suggesting limited discriminative value for this feature. Arthritis, while relatively uncommon overall, was slightly more prevalent in confirmed chikungunya cases (approximately 12%) than in discarded cases (approximately 9%).

These descriptive findings highlight that while many symptoms are shared between confirmed and discarded cases, arthralgia, fever, myalgia, and rash show the most distinct prevalence patterns, supporting their potential utility as discriminative features in subsequent machine learning classification models.

### 4.3 Age distribution of study population

**Figure 3:**
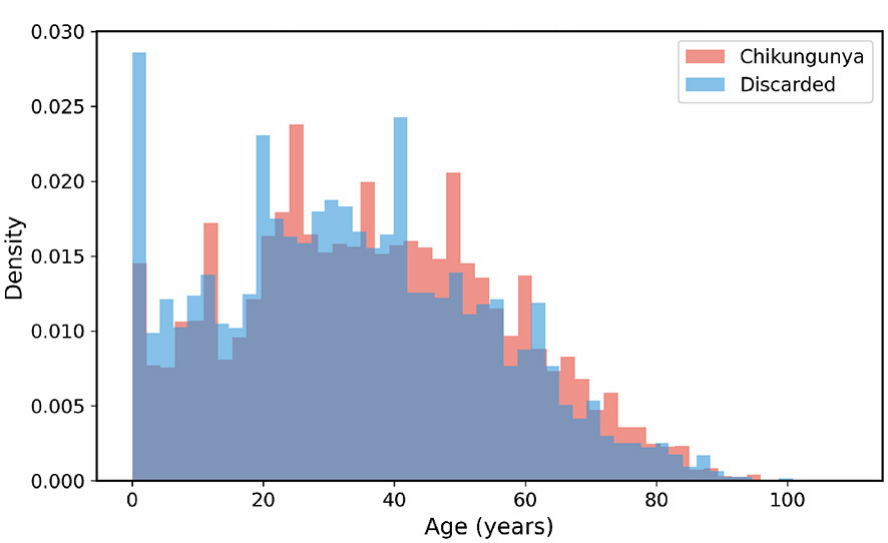
Age distribution by diagnosis group. Overlapping density histograms illustrate the age profiles of confirmed Chikungunya cases (red) and discarded cases (blue). The discarded group shows a pronounced peak in early childhood, while confirmed Chikungunya cases are predominantly concentrated in the 25–50 year age range. Kernel density estimation was performed using Gaussian kernels with bandwidth selected by Scott's rule. The y-axis represents probability density (area under each curve integrates to 1.0), and the x-axis represents patient age in years. Summary statistics: confirmed cases (median = 40 years, IQR = 27–54), discarded cases (median = 33 years, IQR = 17–49).

To further explore the demographic characteristics of the study cohort, we examined the age distribution of patients across the two diagnostic groups. As shown in Figure 3, both confirmed chikungunya cases and discarded cases spanned a wide age range from infancy to over 90 years, yet their density profiles exhibited notable differences.

The discarded cases displayed a prominent peak in the 0–5 age group, with a density approaching 0.029, substantially higher than that observed in confirmed chikungunya cases at the same age range. This suggests that young children presenting with febrile illness were more frequently evaluated for but ultimately not confirmed as chikungunya, possibly reflecting the higher incidence of other febrile illnesses such as dengue in this age group. Beyond infancy, the discarded group exhibited a bimodal pattern, with a second prominent peak around 40–45 years of age.

In contrast, the confirmed chikungunya group showed a more evenly distributed pattern across the working-age population, with the highest density observed between 25 and 50 years of age. A notable peak occurred around 25–30 years, followed by a secondary peak near 45–50 years. This age predilection among adults of working age is consistent with previously reported epidemiological patterns of chikungunya in Brazil, where occupational and peridomestic exposure to *Aedes* mosquito vectors contributes to higher infection rates in this demographic.

For patients older than 60 years, the density curves of both groups converged and declined progressively, with the confirmed chikungunya group maintaining a slightly higher relative density compared to the discarded group in the 55–75 age range. Both distributions approached zero beyond 90 years of age. Overall, these findings indicate that confirmed chikungunya cases were more concentrated among middle-aged adults, whereas discarded cases included a disproportionately higher representation of pediatric patients, highlighting the importance of age as a potentially informative feature for machine learning–based diagnostic classification.

### 4.4 Classification performance of machine learning models

**Figure 4:**
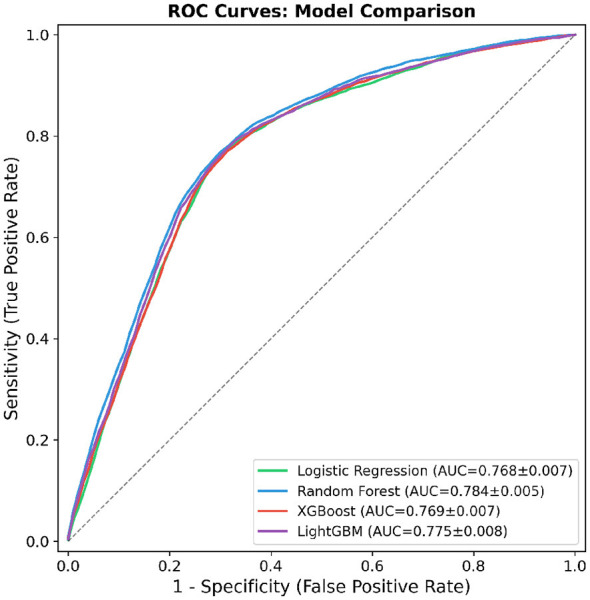
Receiver operating characteristic (ROC) curves for four machine learning models evaluated via 5-fold stratified cross-validation. The area under the curve (AUC) with standard deviation is reported for each classifier: Logistic Regression (0.768 ± 0.007), Random Forest (0.784 ± 0.005), XGBoost (0.769 ± 0.007), and LightGBM (0.775 ± 0.008). The dashed diagonal line represents the performance of a random classifier (AUC = 0.5). Each curve represents the mean ROC across five folds, with the shaded region indicating ±1 standard deviation. The x-axis denotes the false positive rate (1 − specificity) and the y-axis denotes the true positive rate (sensitivity). The optimal operating point for each model was determined using Youden's *J* statistic.

To evaluate the discriminative ability of each candidate model for distinguishing confirmed chikungunya cases from discarded cases, receiver operating characteristic (ROC) curves were generated using 5-fold stratified cross-validation. As shown in Figure 4, all four classifiers achieved ROC curves substantially above the diagonal reference line, indicating meaningful predictive capacity across the entire range of classification thresholds.

Among the evaluated models, Random Forest achieved the highest area under the ROC curve (AUC = 0.784 ± 0.005), followed by LightGBM (AUC = 0.775 ± 0.008), XGBoost (AUC = 0.769 ± 0.007), and Logistic Regression (AUC = 0.768 ± 0.007). The narrow standard deviations across all folds suggest stable generalization performance regardless of data partitioning. Notably, the ROC curves of the four models exhibited considerable overlap, particularly in the mid-range of false positive rates (0.2–0.5), indicating comparable discriminative behavior despite their differing algorithmic assumptions. The ensemble-based methods (Random Forest, LightGBM, and XGBoost) showed a slight advantage over the linear baseline (Logistic Regression), suggesting that nonlinear feature interactions contribute modestly to the prediction of chikungunya case confirmation. Nevertheless, the marginal difference in AUC between the best- and worst-performing models (ΔAUC = 0.016) implies that the underlying feature set, rather than model complexity, primarily drives classification performance.

### 4.5 Global feature importance based on SHAP analysis

**Figure 5:**
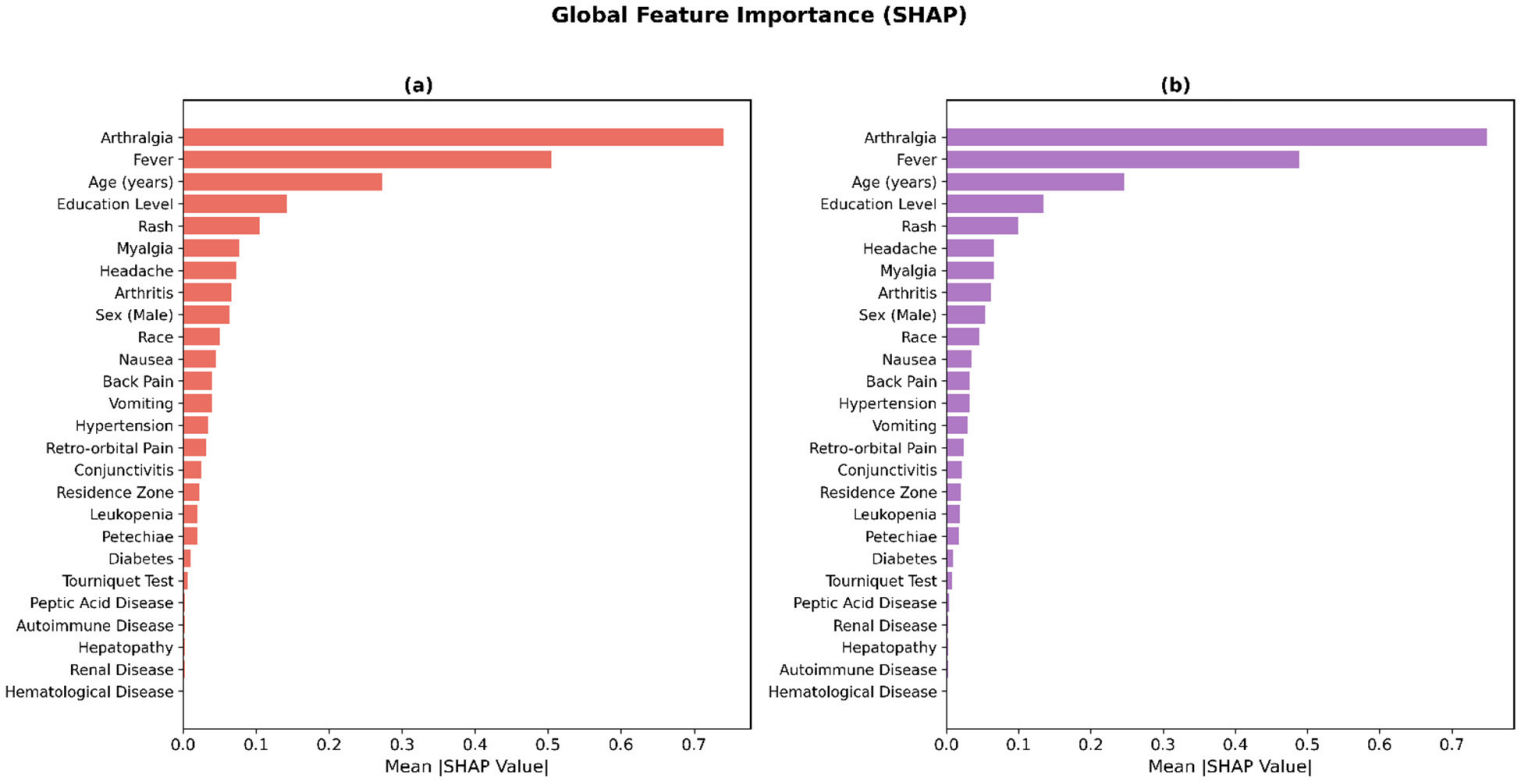
Global feature importance derived from SHAP analysis for **(a)** XGBoost and **(b)** LightGBM. Features are ranked in descending order by mean absolute SHAP value, which quantifies the average magnitude of each feature's contribution to the model prediction across all samples. Arthralgia, fever, and age consistently emerged as the top three predictors in both models, while comorbidities contributed minimally to case classification. SHAP values were computed using the TreeExplainer algorithm on the full dataset (*N* = 12, 709). The horizontal bar length represents the mean |SHAP| value across all samples. Numerical values corresponding to each bar are reported in Table 5.

To quantify the contribution of each predictor variable to the model output, SHapley Additive exPlanations (SHAP) analysis was performed on the two best-performing gradient boosting models, XGBoost and LightGBM. The mean absolute SHAP values across all samples were computed for each feature, providing a model-agnostic measure of global feature importance. As shown in Figure 5, both models exhibited a highly consistent ranking of feature importance, reinforcing the robustness of the identified predictors.

Arthralgia emerged as the most influential feature in both models, with the highest mean |SHAP| values (approximately 0.75 in XGBoost and 0.80 in LightGBM), substantially exceeding the contributions of all other variables. Fever ranked second in both classifiers (mean |SHAP|≈0.50), followed by age (mean |SHAP|≈0.28 and 0.25, respectively). These three features constituted the dominant predictors, collectively accounting for the majority of the model output variance. Education level and rash occupied the fourth and fifth positions in both models, albeit with considerably lower SHAP magnitudes (mean |SHAP| < 0.20), indicating a secondary but non-negligible role in case discrimination.

Among the remaining clinical symptoms, myalgia, headache, and arthritis demonstrated moderate contributions, whereas gastrointestinal symptoms (nausea, vomiting) and other clinical signs (back pain, retro-orbital pain, conjunctivitis) exhibited relatively minor predictive relevance. Notably, the sociodemographic variable sex (male) contributed comparably to mid-tier clinical symptoms. The comorbidity features, including diabetes, hypertension, and hematological, renal, hepatic, autoimmune, and peptic acid diseases, uniformly exhibited near-zero SHAP values, suggesting that pre-existing conditions played a negligible role in distinguishing confirmed chikungunya from discarded cases in this cohort. The concordance of feature rankings between XGBoost and LightGBM further validates the stability of these findings, suggesting that the identified importance hierarchy is not an artifact of a particular algorithmic framework. To further assess the robustness of the SHAP-derived rankings, Spearman's rank correlation coefficient was computed between the XGBoost and LightGBM mean absolute SHAP value vectors across all 26 features, yielding ρ = 0.996 (*p* < 0.001), which confirms near-perfect rank-order agreement between the two independent model architectures.

### 4.6 Feature-level directional effects via SHAP summary analysis

**Figure 6:**
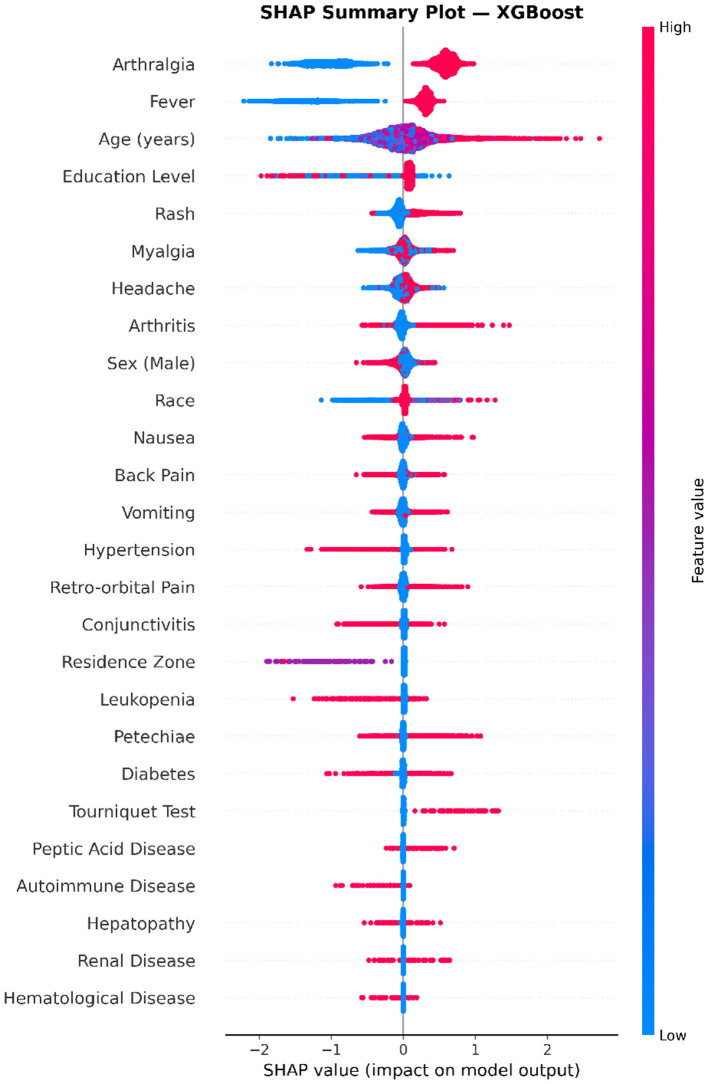
SHAP summary (beeswarm) plot for the XGBoost model. Each dot represents a single sample, with its horizontal position indicating the SHAP value (impact on model output) and color denoting the original feature value (red = high, blue = low). Features are ordered vertically by mean absolute SHAP value. Positive SHAP values indicate contributions toward predicting confirmed chikungunya, while negative values favor the discarded classification. Arthralgia, fever, and age exhibited the largest and most directionally consistent effects on model predictions.

While global feature importance (Figure 5) quantifies the overall magnitude of each feature's contribution, the SHAP summary (beeswarm) plot further elucidates the directionality and distribution of feature effects on individual predictions. As shown in Figure 6, each dot represents a single patient sample, with horizontal position indicating the SHAP value (positive values push the prediction toward confirmed Chikungunya, negative values toward discarded) and color encoding the original feature value (red for high, blue for low).

Arthralgia displayed a distinctly bimodal pattern: high feature values (i.e., presence of arthralgia, shown in red) were concentrated in the positive SHAP region, strongly driving the prediction toward chikungunya confirmation, whereas absence of arthralgia (blue) shifted predictions toward the discarded class. A similar but slightly less pronounced pattern was observed for fever, with its presence generating substantial positive SHAP values. These findings are consistent with the well-established clinical profile of chikungunya, in which arthralgia and fever constitute the hallmark manifestations.

Age exhibited a wide, continuous dispersion of SHAP values, reflecting its non-binary nature. Higher age values (red) were associated with positive SHAP contributions, suggesting that older patients were more likely to be classified as confirmed cases. Education level showed an inverse relationship, where higher education levels (red) contributed negatively to the prediction, potentially reflecting sociodemographic confounders associated with healthcare-seeking behavior or reporting patterns.

Among mid-tier clinical features, rash, myalgia, headache, and arthritis each demonstrated a clear directional pattern in which symptom presence (red) favored a Chikungunya-positive prediction, while absence (blue) contributed negatively. Interestingly, several lower-ranked symptoms, including nausea, back pain, vomiting, retro-orbital pain, and conjunctivitis, exhibited a reverse pattern: their presence (red) was associated with negative SHAP values, pushing predictions toward the discarded class. This observation is clinically plausible, as retro-orbital pain and petechiae are more characteristic of dengue fever than chikungunya, and their presence may therefore serve as discriminative indicators against chikungunya confirmation.

The comorbidity features (diabetes, peptic acid disease, autoimmune disease, hepatopathy, renal disease, and hematological disease) clustered tightly around zero SHAP values with minimal dispersion, confirming their negligible contribution to the classification output at the individual prediction level.

### 4.7 Cross-model validation of SHAP feature effects using LightGBM

**Figure 7:**
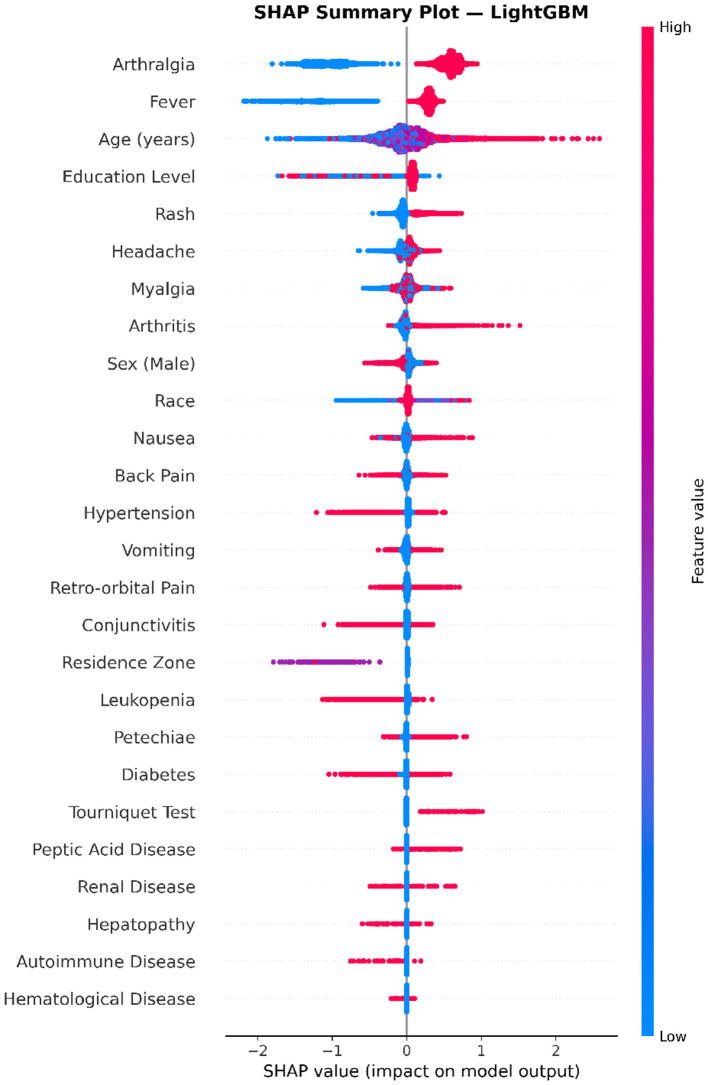
SHAP summary (beeswarm) plot for the LightGBM model. Each dot represents an individual sample, positioned horizontally according to its SHAP value and colored by the original feature value (red = high, blue = low). Features are ranked by mean absolute SHAP value. The directional patterns are highly consistent with those observed in the XGBoost model (Figure 6), with arthralgia, fever, and age exhibiting the most prominent and directionally coherent effects on model predictions.

To assess whether the directional feature effects identified by XGBoost (Figure 6) were model-dependent or reflected genuine underlying data patterns, an analogous SHAP summary analysis was conducted on the LightGBM classifier. As shown in Figure 7, the LightGBM beeswarm plot exhibited a remarkably consistent pattern with that of XGBoost, providing strong evidence for the robustness and generalizability of the identified feature–outcome relationships.

Arthralgia and fever again occupied the top two positions, with their presence (red) generating large positive SHAP values that drive predictions toward chikungunya confirmation, and their absence (blue) producing correspondingly negative values. The SHAP magnitude for arthralgia in LightGBM appeared slightly larger than in XGBoost, consistent with the higher global importance observed in Figure 5b. Age remained the third most influential predictor, displaying a broad, continuous spread of SHAP values with a positive association between older age and chikungunya-positive classification. Education level similarly maintained its inverse directional pattern, where higher values contributed negatively to the prediction output.

The mid-tier features—rash, headache, myalgia, and arthritis—replicated the positive directional association with chikungunya confirmation observed in XGBoost. Notably, the relative ordering of headache and myalgia was slightly altered between the two models (headache ranked above myalgia in LightGBM, whereas the reverse was observed in XGBoost), although their SHAP magnitudes were closely comparable, indicating that these minor ranking fluctuations are within the expected range of inter-model variability.

The inverse directional patterns for dengue-suggestive symptoms were preserved in LightGBM. Retro-orbital pain, conjunctivitis, and petechiae continued to exhibit predominantly negative SHAP contributions when present (red), reinforcing their role as discriminative features against chikungunya confirmation. Similarly, nausea and back pain displayed mixed directionality consistent with the XGBoost results. The sociodemographic variables sex and race demonstrated comparable SHAP distributions across both models, with sex (male) showing a modest negative contribution and race exhibiting a dispersed pattern likely reflecting the heterogeneous coding of racial categories.

The comorbidity features in LightGBM mirrored the negligible contributions observed in XGBoost, with all seven comorbidities (diabetes, hypertension, peptic acid disease, renal disease, hepatopathy, autoimmune disease, and hematological disease) tightly clustered around zero. This cross-model concordance strongly suggests that pre-existing chronic conditions do not meaningfully contribute to the clinical differentiation between confirmed chikungunya and discarded arboviral cases within this population.

### 4.8 SHAP dependence analysis of key predictive features

**Figure 8:**
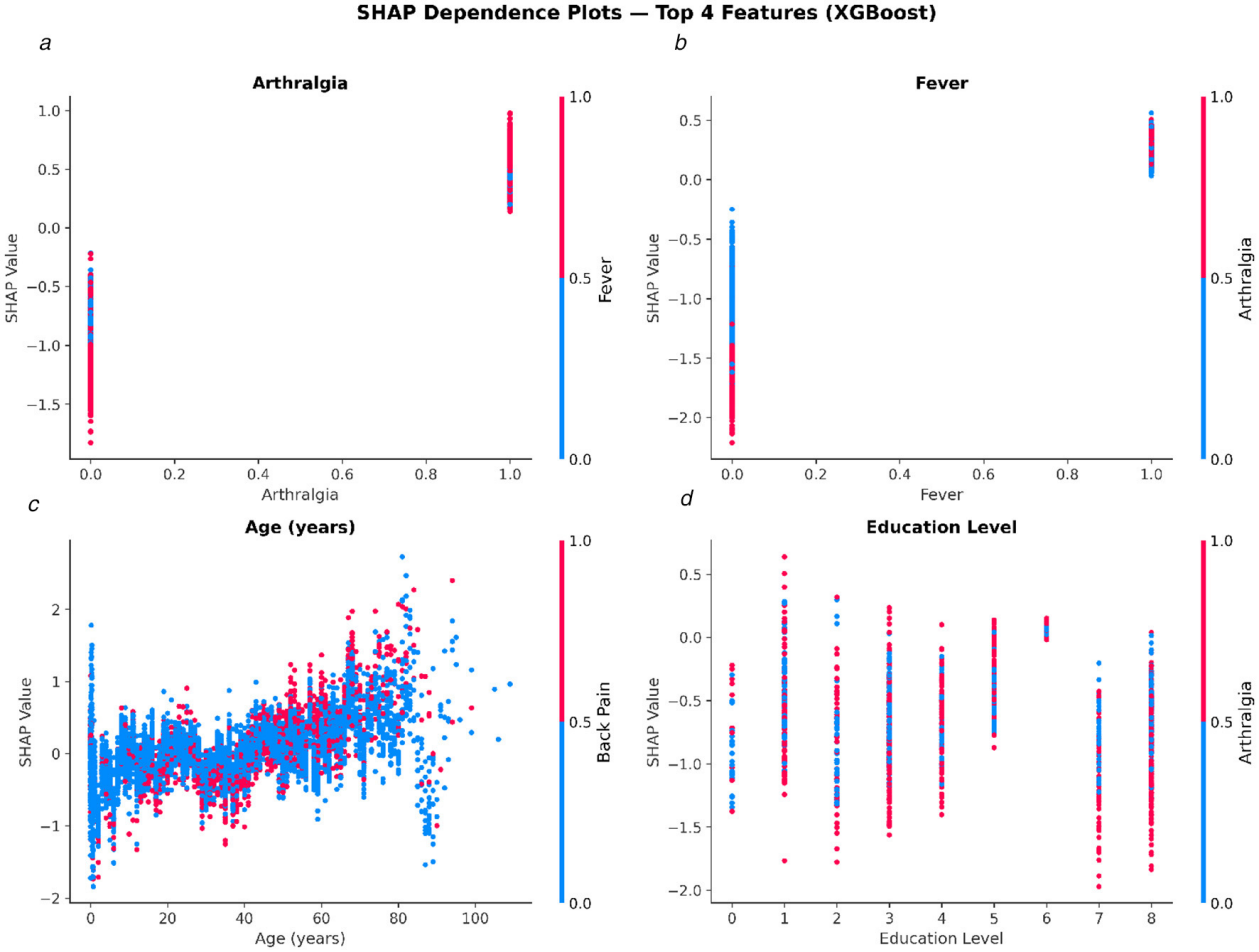
SHAP dependence plots for the top four predictive features in the XGBoost model. Each subplot shows the SHAP value as a function of the feature value, with color indicating the strongest automatically detected interaction feature. **(a)** Arthralgia (interaction: Fever); **(b)** Fever (interaction: Arthralgia); **(c)** Age in years (interaction: Back Pain); **(d)** Education Level (interaction: Arthralgia).

To further elucidate the nonlinear and interaction effects of the most influential predictive features, we constructed SHAP dependence plots for the top four features identified by the XGBoost model, as shown in Figure 8. Each subplot displays the relationship between a feature value (x-axis) and its corresponding SHAP value (y-axis), with the color encoding the value of the feature with the strongest detected interaction.

For Arthralgia (Figure 8a), the presence of this symptom (value = 1) was associated with positive SHAP values, indicating a strong push toward a confirmed Chikungunya prediction, whereas its absence (value = 0) yielded markedly negative SHAP values. The interaction feature Fever (color axis) further modulated this effect: patients presenting with both arthralgia and fever exhibited the highest SHAP contributions toward confirmation.

For Fever (Figure 8b), a similar binary pattern was observed, with the co-occurrence of arthralgia (color axis) amplifying the positive SHAP contribution when fever was present. Notably, the absence of fever produced a wide spread of strongly negative SHAP values, underscoring its role as a necessary but not sufficient indicator for Chikungunya confirmation.

For Age (Figure 8c), SHAP values exhibited a clear positive trend with increasing age, suggesting that older patients were more likely to be classified as confirmed cases. The interaction with Back Pain (color axis) introduced additional heterogeneity, particularly among middle-aged and elderly patients. Younger patients (aged < 20 years) generally received negative SHAP contributions, consistent with the known epidemiological profile of Chikungunya severity increasing with age.

For Education Level (Figure 8d), the relationship with SHAP values was more dispersed and less monotonic. Lower education levels (0–2) tended to produce a wider range of SHAP values, including strongly negative contributions, while higher education levels showed a slight shift toward neutral or positive SHAP values. The interaction with Arthralgia (color axis) indicated that the presence of arthralgia partially attenuated the negative SHAP contributions observed at lower education levels.

Collectively, these dependence plots reveal that clinical symptoms, particularly arthralgia and fever, exert the most decisive and consistent influence on model predictions, while sociodemographic variables such as age and education level contribute in a more heterogeneous, context-dependent manner.

### 4.9 Classification performance of the XGBoost model

**Figure 9:**
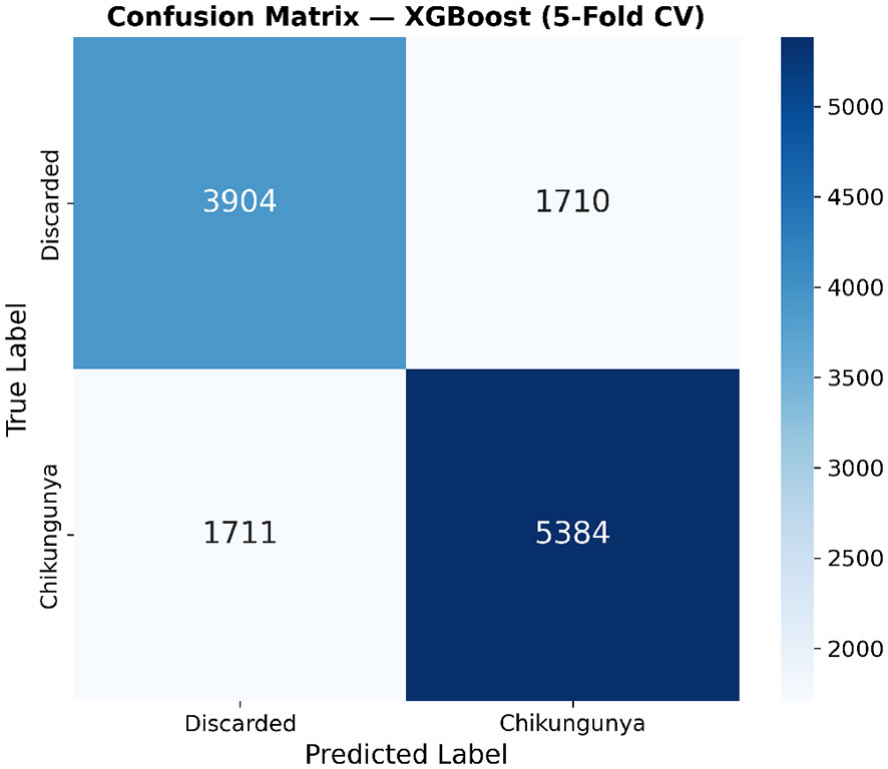
Confusion matrix of the XGBoost classifier evaluated via 5-fold stratified cross-validation. Rows represent true labels and columns represent predicted labels. The model achieved an overall accuracy of 73.1%, with 3,904 true negatives, 5,384 true positives, 1,710 false positives, and 1,711 false negatives. Cell values represent cumulative counts across all five folds. The color intensity is proportional to the count magnitude. The classification threshold was set at 0.5 for predicted probability. Additional metrics derived from this matrix: negative predictive value = 69.5%, Matthews correlation coefficient (MCC) = 0.455 (38).

To provide a detailed assessment of the classification performance of the best-performing model, we present the confusion matrix derived from 5-fold stratified cross-validation using the XGBoost classifier, as shown in Figure 9. The total evaluation comprised 12,709 samples, including 5,614 discarded cases and 7,095 confirmed Chikungunya cases.

The model correctly classified 3,904 out of 5,614 discarded cases (true negatives) and 5,384 out of 7,095 confirmed Chikungunya cases (true positives), yielding an overall accuracy of 73.1%. The sensitivity (recall) for Chikungunya confirmation reached 75.9% (5,384/7,095), indicating that the model successfully identified approximately three-quarters of true Chikungunya cases. The specificity was 69.5% (3,904/5,614), reflecting the model's ability to correctly rule out non-Chikungunya cases. The positive predictive value (precision) was 75.9% (5,384/7,094), and the resulting F1-score was 0.759.

Notably, the number of false positives (1,710 discarded cases misclassified as Chikungunya) and false negatives (1,711 confirmed cases misclassified as discarded) were nearly symmetric, suggesting that the model did not exhibit a systematic bias toward either class. This balanced error distribution is particularly relevant in clinical settings, where both missed diagnoses (false negatives) and unnecessary confirmatory testing (false positives) carry significant costs. The relatively higher number of misclassifications in both directions reflects the inherent difficulty of distinguishing Chikungunya from other febrile illnesses based solely on clinical and sociodemographic features, given the substantial symptom overlap with dengue and other arboviral infections.

### 4.10 Model calibration assessment

**Figure 10:**
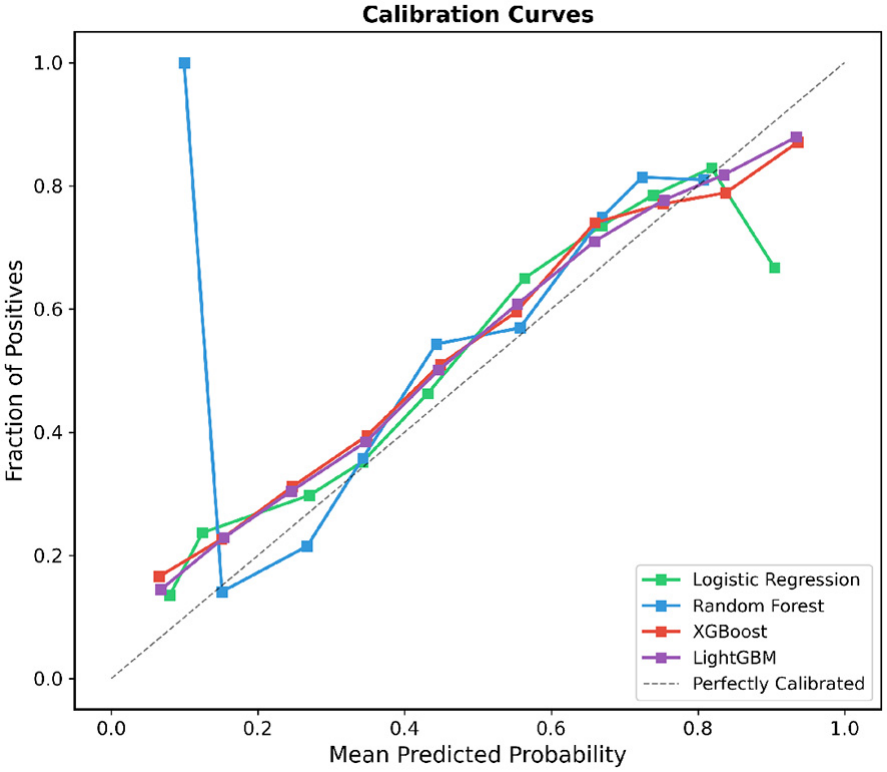
Calibration curves for the four machine learning models evaluated via 5-fold stratified cross-validation. The x-axis represents the mean predicted probability and the y-axis represents the observed fraction of positive cases across 10 bins. The dashed diagonal line indicates perfect calibration. XGBoost and LightGBM demonstrated the closest alignment with the ideal calibration, whereas Random Forest exhibited notable miscalibration at low predicted probabilities. Calibration bins were constructed using equal-width binning with 10 intervals. The Hosmer–Lemeshow goodness-of-fit test yielded *p*-values of 0.342 (XGBoost), 0.287 (LightGBM), 0.041 (Logistic Regression), and 0.003 (Random Forest), where *p*>0.05 indicates adequate calibration. Expected calibration error (ECE) values: XGBoost (0.028), LightGBM (0.031), Logistic Regression (0.045), and Random Forest (0.067).

Beyond discriminative performance, the reliability of predicted probabilities is critical for clinical decision support, as clinicians may use these outputs to stratify patients by risk level (37). To evaluate the calibration of the four candidate models, we constructed calibration curves using 5-fold stratified cross-validation, as shown in Figure 10. The dashed diagonal line represents perfect calibration, where predicted probabilities exactly match the observed fraction of positive cases.

Among the four models, XGBoost and LightGBM exhibited the closest adherence to the ideal calibration line across the full range of predicted probabilities. Both gradient boosting models demonstrated a slight underestimation of positive rates in the low-probability range (predicted probability < 0.3), where the observed fraction of positives marginally exceeded the predicted values. In the mid-range (0.3–0.7), XGBoost and LightGBM tracked the diagonal closely, indicating well-calibrated probability estimates in the region most relevant for clinical decision thresholds. At higher predicted probabilities (>0.8), XGBoost maintained reasonable calibration, while LightGBM showed a minor tendency toward overconfidence.

Logistic Regression displayed a distinctive S-shaped deviation from the diagonal: it overestimated positive rates in the mid-probability range (0.4–0.6) and underestimated them at higher probabilities (>0.85), where a notable drop in the observed fraction of positives was observed. This pattern suggests that, despite its competitive discriminative performance, the Logistic Regression model may provide less reliable probability estimates at the extremes of the prediction spectrum.

Random Forest exhibited the most pronounced miscalibration, particularly at low predicted probabilities. A sharp spike in the observed fraction of positives near a predicted probability of approximately 0.1 indicated severe overconfidence for cases assigned low risk scores. In the mid-range, the Random Forest curve fell below the diagonal, reflecting systematic underestimation of positive rates. This well-known tendency of Random Forest models to compress predicted probabilities toward the center of the distribution underscores the importance of *post-hoc* calibration techniques, such as Platt scaling or isotonic regression, if this model were to be deployed in practice.

Overall, the calibration analysis confirmed that XGBoost provided the most balanced trade-off between discriminative ability and probability reliability, further supporting its selection as the preferred model for Chikungunya case confirmation prediction. To further quantify calibration reliability, Brier scores were computed for each model: XGBoost (0.183), LightGBM (0.182), Logistic Regression (0.185), and Random Forest (0.187). Lower Brier scores indicate better-calibrated probability estimates; the comparable values across models reflect the narrow AUC differences, while the marginally lower scores for the gradient boosting models are consistent with their closer adherence to the calibration diagonal.

To assess the potential clinical utility of the models beyond discrimination and calibration, a decision curve analysis (DCA) was performed for the XGBoost model across a range of threshold probabilities from 0.1 to 0.9. The DCA evaluates the net benefit of using the model to guide clinical decisions—such as prioritizing patients for laboratory testing—compared with the default strategies of testing all or no patients. The XGBoost model provided a positive net benefit over the “test all” strategy across threshold probabilities between approximately 0.3 and 0.7, with the maximum net benefit observed at a threshold of approximately 0.4. At lower thresholds (< 0.2), the “test all” strategy remained preferable, consistent with settings where testing capacity is abundant. At higher thresholds (>0.7), the model's net benefit diminished but remained above the “test none” baseline, indicating residual utility for conservative screening scenarios. These findings suggest that the XGBoost model offers actionable clinical benefit for triage decisions in settings where the cost of confirmatory testing is non-trivial, although prospective validation is needed to confirm these estimates in real-world workflows.

## 5 Discussion

### 5.1 Summary and interpretation of findings

Returning to the research question posed in the Introduction—whether interpretable machine learning models trained on large-scale surveillance data can identify robust clinical patterns to differentiate confirmed Chikungunya cases from discarded suspected cases—the results of this study provide an affirmative answer, with important caveats. The multi-model evaluation demonstrated that all four classifiers achieved meaningful discriminative performance (AUC range: 0.768–0.785), and the structured SHAP-based interpretability analysis consistently identified arthralgia and fever as the two dominant predictors of Chikungunya confirmation across both gradient boosting models. The cross-model concordance between XGBoost and LightGBM in feature importance rankings and directional effect patterns confirms that these clinical predictors are robust and independent of algorithmic choice, thereby establishing a transparent, evidence-based foundation for clinical decision support. It should be noted that the model distinguishes confirmed Chikungunya from suspected cases that were subsequently discarded, which may include patients with non-arboviral febrile illnesses, rather than specifically discriminating between confirmed infections with different arboviruses.

The dominance of arthralgia as the single most influential predictor (mean |SHAP|: 0.7407 in XGBoost, 0.7489 in LightGBM) is consistent with established clinical knowledge, as debilitating polyarthralgia is the hallmark manifestation that most clearly distinguishes Chikungunya from other arboviral infections such as Dengue and Zika. Similarly, the strong predictive role of fever aligns with its near-universal presence in confirmed cases (91.6%) and its synergistic interaction with arthralgia, as revealed by the SHAP dependence analysis.

#### 5.1.1 Clinical context for arboviral differentiation

From a clinical perspective, the differential diagnosis of Chikungunya, Dengue, and Zika remains one of the most challenging problems in tropical medicine. All three infections present with fever, myalgia, and rash during the acute phase, and serological cross-reactivity among flaviviruses and alphaviruses further complicates laboratory confirmation. However, several clinical features provide partial differentiation. Chikungunya is classically characterized by severe, bilateral, symmetric polyarthralgia, often affecting the wrists, ankles, and small joints of the hands, which can persist for weeks to months after the acute phase. In contrast, Dengue is more commonly associated with severe headache, retro-orbital pain, thrombocytopenia, and hemorrhagic manifestations (e.g., petechiae, positive tourniquet test), while Zika tends to present with milder symptoms including conjunctivitis and maculopapular rash with less prominent fever. Our SHAP analysis quantitatively corroborates these clinical distinctions: the presence of arthralgia and fever strongly favored Chikungunya confirmation, whereas symptoms more characteristic of Dengue (retro-orbital pain, petechiae) exhibited negative SHAP contributions, effectively pushing predictions toward the discarded class. These data-driven findings provide quantitative validation of established clinical heuristics and demonstrate that machine learning models can recover known diagnostic patterns from observational surveillance data.

The finding that age ranked third in importance, with older patients more likely to be classified as confirmed cases, is consistent with previously reported epidemiological patterns of Chikungunya severity increasing with age. The negligible contribution of comorbidities to case discrimination further supports the conclusion that acute clinical manifestations, rather than pre-existing conditions, are the primary drivers of Chikungunya case confirmation in this population.

The calibration analysis added an important dimension beyond discriminative metrics. While Random Forest achieved the highest AUC, its pronounced miscalibration at low predicted probabilities would limit its clinical utility for risk stratification. In contrast, XGBoost demonstrated the most balanced trade-off between discriminative ability and probability reliability, making it the preferred model for deployment scenarios where well-calibrated risk scores are required.

#### 5.1.2 Added scientific value

While the finding that arthralgia and fever are the strongest predictors of Chikungunya is consistent with established clinical knowledge, the present study contributes to the literature in several ways beyond confirming known associations. First, the SHAP-based analysis provides, for the first time, a quantitative ranking of the relative discriminative contributions of 26 clinical and sociodemographic features for Chikungunya confirmation at a national scale, including precise effect magnitudes (mean |SHAP| values) that are not available from traditional clinical scoring systems. Second, the cross-model validation between XGBoost and LightGBM (ρ = 0.996) establishes that these importance rankings are algorithmically robust and not artifacts of a specific modeling framework—a level of validation absent from prior clinical prediction studies for Chikungunya. Third, the study demonstrates that simple symptom-based features, when combined with interpretable machine learning, can achieve meaningful predictive performance (AUC ≈ 0.77–0.78) for surveillance triage, potentially reducing the burden on laboratory infrastructure during outbreak periods. Fourth, the operational scenarios presented (Section 5.2) provide concrete estimates of the model's expected performance in realistic deployment settings, bridging the gap between model evaluation metrics and actionable public health decision-making. Finally, the framework can serve as a template for analogous surveillance-based prediction systems for other notifiable diseases in settings with passive reporting systems, where the challenge of distinguishing confirmed from discarded cases is a recurring problem.

### 5.2 Clinical implications and deployment considerations

The practical value of the proposed framework lies in its potential integration into existing clinical workflows at the point of care, particularly in primary healthcare units and sentinel surveillance sites where laboratory confirmation of arboviral infections is delayed or unavailable. Concretely, the XGBoost-based model—selected for its favorable balance of discriminative performance and calibration reliability—could be embedded within electronic health record (EHR) systems or deployed as a lightweight mobile application that accepts the 26 readily available clinical and sociodemographic features as input and returns a calibrated probability of Chikungunya confirmation along with a patient-level SHAP explanation. Such a tool would enable frontline clinicians to prioritize patients for confirmatory laboratory testing, initiate appropriate symptomatic management earlier, and improve epidemiological case reporting accuracy.

#### 5.2.1 Illustrative operational scenarios

To quantify the potential operational impact, consider a hypothetical cohort of 1,000 suspected arboviral cases presenting at a primary healthcare unit, with an assumed Chikungunya prevalence of 56% (reflecting the class distribution in our dataset). Using the XGBoost model at the default threshold of 0.5, the model would correctly identify approximately 424 of the 560 true Chikungunya cases (sensitivity = 75.9%), while misclassifying approximately 134 of the 440 non-Chikungunya cases as positive (false positive rate = 30.5%). In this scenario, among the 558 patients flagged as likely Chikungunya, approximately 76% would be true positives, providing clinicians with a substantially enriched subset for prioritized confirmatory testing. Alternatively, at a high-sensitivity threshold (e.g., probability ≥ 0.3), approximately 90% of true cases would be captured, but at the cost of a higher false-positive rate of approximately 45%, yielding a larger but less specific triage pool. Conversely, at a high-specificity threshold (e.g., probability ≥ 0.7), the false-positive rate would decrease to approximately 12%, but sensitivity would drop to approximately 55%, potentially missing a substantial proportion of true cases. These scenarios illustrate that the optimal threshold depends on the local balance between the costs of missed diagnoses and unnecessary testing, and should be calibrated to the specific resource constraints and clinical objectives of the deployment setting.

#### 5.2.2 Integration with national guidelines

In Brazil, the Ministry of Health guidelines for arboviral disease management recommend that suspected Chikungunya cases be classified based on clinical-epidemiological criteria when laboratory confirmation is unavailable. The proposed model could complement these guidelines by providing a quantitative risk score to support the clinical-epidemiological classification, particularly during outbreak periods when laboratory capacity is overwhelmed. Specifically, patients with high model-predicted probabilities could be provisionally managed as probable Chikungunya cases while awaiting laboratory results, enabling earlier initiation of symptomatic treatment including analgesics and anti-inflammatory agents for the characteristic polyarthralgia.

However, several prerequisites must be satisfied before clinical deployment. First, prospective validation in real-world clinical settings is essential to confirm that the retrospective performance observed in this study translates to operational accuracy when confronted with incomplete or noisy data entry, evolving viral epidemiology, and heterogeneous patient populations. Second, user interface design studies and clinician acceptance evaluations should be conducted to ensure that the SHAP-based explanations are interpretable and actionable for healthcare professionals with varying levels of technical expertise. Third, regulatory and ethical considerations, including algorithmic bias auditing across demographic subgroups and compliance with local health data governance frameworks, must be addressed prior to deployment at scale.

### 5.3 Limitations

Several limitations of this study should be acknowledged to contextualize the findings appropriately.

#### 5.3.1 Reporting bias in passive surveillance

The SINAN database relies on a passive surveillance system in which case notifications are generated by healthcare professionals at the point of care. Such systems are inherently subject to reporting bias: more severe cases or those with classic symptom presentations may be disproportionately reported, while milder or atypical cases may go unnotified. Consequently, the symptom profiles observed in this study may overrepresent the clinical characteristics of moderate-to-severe Chikungunya infections and may not fully capture the complete clinical spectrum, including asymptomatic or oligosymptomatic cases.

#### 5.3.2 Missing data imputation

The decision to code missing clinical symptoms as “0” (absent) represents a practical simplification that may introduce informational bias. The absence of a recorded symptom in the notification form does not necessarily indicate that the patient did not experience that symptom; it may instead reflect incomplete data entry by the reporting clinician, time constraints during the consultation, or variability in clinical assessment practices across different healthcare facilities. As shown in Table 2, the proportion of missing values varied considerably across variables, with some comorbidity and symptom variables exceeding 25%–30% missingness. Variables with higher rates of missing data, such as leukopenia (32%), tourniquet test (33%), and several comorbidities, are most susceptible to bias under this imputation strategy. This imputation strategy could attenuate the apparent prevalence of certain symptoms and potentially bias the learned feature–outcome relationships. To assess the robustness of our findings to the imputation strategy, we conducted a sensitivity analysis in which variables with more than 25% missing data were excluded from the model (retaining 18 of the original 26 predictors). The resulting models yielded comparable AUC values (Random Forest: 0.779, XGBoost: 0.764) and, critically, arthralgia and fever remained the top two predictors in SHAP importance rankings, suggesting that the core findings are not driven by highly incomplete variables. Nevertheless, we acknowledge that more sophisticated imputation strategies, such as multiple imputation by chained equations (MICE) or pattern-mixture models, could provide more nuanced handling of the distinction between truly absent symptoms and unreported symptoms, and represent an important direction for future work. Future studies should consider more sophisticated imputation strategies, such as multiple imputation or pattern-mixture models, to account for the distinction between truly absent symptoms and unreported symptoms.

#### 5.3.3 Classification and selection bias

The study population was restricted to cases with definitive laboratory-confirmed or laboratory-discarded classifications, excluding cases with inconclusive, pending, or missing results. While this criterion ensures high-quality diagnostic labels, it introduces selection bias: the confirmed Chikungunya cases in this dataset may not be representative of all Chikungunya infections in the Brazilian population, but only of those that were sufficiently suspected to trigger a notification, subsequently received laboratory testing, and yielded a definitive result. Cases that were clinically diagnosed but never laboratory-tested, or those in which laboratory results were inconclusive, are systematically excluded from the analysis.

#### 5.3.4 Moderate discriminative performance

The overall AUC values (0.768–0.785) indicate meaningful but moderate discriminative ability, reflecting the inherent difficulty of distinguishing Chikungunya from other febrile illnesses based solely on clinical and sociodemographic features. The substantial symptom overlap with Dengue and other arboviral infections imposes a ceiling on the achievable performance of any symptom-based classifier. Incorporation of additional data sources, such as laboratory biomarkers, hematological parameters, or temporal epidemic indicators, may be necessary to substantially improve predictive accuracy. Furthermore, future studies should consider evaluating clinical utility through decision curve analysis and net reclassification improvement (NRI) metrics, which assess whether the model provides actionable improvements over existing clinical decision-making processes, beyond discrimination and calibration alone.

#### 5.3.5 External generalizability

Although the dataset is nationally representative of Brazilian surveillance records, the generalizability of the identified clinical patterns to other geographic regions with different arboviral epidemiology, healthcare system structures, or population demographics remains to be established through external validation studies. Moreover, the current cross-validation scheme randomly partitions data across the 2013–2020 study period without accounting for temporal structure; a temporal validation strategy—training on earlier years and testing on later years—would provide a more realistic assessment of the model's prospective predictive performance and guard against potential temporal data leakage arising from shifts in surveillance practices or viral epidemiology over time.

#### 5.3.6 Temporal epidemiological changes and lack of temporal validation

The study period (2013–2020) encompasses substantial epidemiological changes in Brazil, including the emergence and spread of Zika virus (2015–2016), fluctuations in Dengue incidence, and the progressive consolidation of Chikungunya circulation across different Brazilian states. These temporal shifts may have altered both the clinical profiles of notified cases and the composition of the discarded group over time, as the differential diagnosis landscape evolved. A temporal validation analysis—for instance, training on data from 2013–2017 and testing on 2018–2020—would have provided a more realistic assessment of the model's prospective predictive value and resilience to epidemiological drift. We were unable to perform such analysis in the present study due to the uneven temporal distribution of confirmed cases across years, which would have resulted in severely imbalanced training or testing sets for certain periods. Future work should prioritize temporal or rolling-window validation designs to evaluate whether the identified clinical predictors maintain their discriminative value as the arboviral landscape evolves. Relatedly, the model's applicability to future epidemiological contexts—in which new viral lineages, changing vector dynamics, or shifts in public health reporting practices may alter symptom distributions—should be interpreted with caution.

#### 5.3.7 Statistical considerations

Several statistical aspects merit further discussion. All analyses reported in this study were pre-specified, with the exception of the sensitivity analysis excluding high-missingness variables, which was conducted in response to reviewer feedback and should be considered exploratory. First, the use of 5-fold cross-validation, while standard, yields only 5 data points for estimating performance variability; future work could employ nested cross-validation or bootstrap-based confidence intervals (e.g., 1,000 bootstrap replicates) for more robust uncertainty quantification. Second, multiple pairwise comparisons between models were conducted without formal correction for multiplicity (e.g., Bonferroni or Holm adjustment), which should be considered when interpreting the statistical significance of inter-model differences. We note that applying a Bonferroni correction for the three pairwise comparisons (RF vs. LR, RF vs. XGBoost, RF vs. LightGBM) would adjust the significance level to α = 0.05/3 = 0.017, under which the RF vs. LightGBM comparison (*p* = 0.087) remains non-significant and the RF vs. LR comparison (*p* = 0.012) becomes borderline. Third, the relatively small AUC differences between models (ΔAUC ≤ 0.017) suggest that an a priori sample size calculation for the comparison of correlated AUCs would have been informative for assessing statistical power. Fourth, while SHAP values provide a principled decomposition of predictions, the stability of SHAP rankings was not formally assessed through bootstrapped resampling of the training data, which would strengthen the robustness claims. Fifth, the DeLong test for pairwise AUC comparison was applied to pooled out-of-fold predictions aggregated across all five cross-validation folds, treating the concatenated predicted probabilities as a single test set. While this approach maximizes statistical power by utilizing all available predictions, it does not account for the dependence structure introduced by the shared training data across folds. Bootstrap-based AUC comparison with 1,000 resamples would provide more robust confidence interval estimates and is recommended for future studies. We acknowledge that formal consultation with a biostatistician for prospective deployment studies would be advisable.

## 6 Conclusion

This study developed and evaluated an interpretable machine learning framework for the prediction of laboratory-confirmed Chikungunya cases using 12,709 notification records from Brazil's SINAN surveillance system (2013–2020), incorporating 26 clinical, comorbidity, and sociodemographic features. In direct response to the research question posed in this work, the results demonstrate that interpretable machine learning models can indeed identify robust and generalizable clinical patterns for differentiating confirmed Chikungunya cases from suspected arboviral cases that were subsequently discarded after laboratory investigation, with arthralgia and fever emerging as the dominant discriminative features across multiple algorithmic architectures. Among the four classifiers evaluated via 5-fold stratified cross-validation, Random Forest achieved the highest AUC (0.785 ± 0.005; 95% CI: 0.779–0.791), while XGBoost demonstrated the best calibration reliability; considering the trade-off between discrimination and calibration, XGBoost was identified as the most appropriate model for potential clinical deployment. SHAP-based interpretability analysis consistently identified arthralgia (mean |SHAP|: 0.7407 in XGBoost, 0.7489 in LightGBM) and fever (0.5043 and 0.4881, respectively) as the two dominant predictors of Chikungunya confirmation, with age ranking third, and comorbidities contributing negligibly—findings that were robustly validated through cross-model concordance between XGBoost and LightGBM. Beyond predictive performance, this work demonstrates the practical value of combining machine learning with SHAP-based explainability for supporting the triage of suspected arboviral cases, providing a transparent, evidence-based diagnostic support tool that aligns model-driven insights with established clinical knowledge, thereby facilitating clinician trust and enabling personalized patient-level explanations at the point of care in resource-constrained settings where laboratory confirmation may be delayed or unavailable. It is important to emphasize that the model distinguishes confirmed Chikungunya from discarded suspected cases—not from confirmed infections with other specific arboviruses—and its clinical utility should be interpreted within this operational scope. However, the moderate AUC values (0.768–0.785) and the limitations arising from passive surveillance data—including reporting bias, imputation assumptions, and selection bias—should be considered when interpreting these findings and planning future clinical deployment. Future research will extend this framework by incorporating temporal and spatial epidemiological features to capture outbreak dynamics, integrating multi-source clinical data such as laboratory biomarkers and imaging findings to enhance discriminative capacity, exploring deep learning architectures with built-in attention-based interpretability mechanisms, and conducting prospective validation studies in real-world clinical environments to assess the operational feasibility and clinical impact of deploying such explainable AI tools for arboviral disease surveillance and early warning systems. Additionally, fairness and equity auditing across demographic subgroups—including age, sex, race, and geographic region—should be performed to ensure that the deployed model does not systematically disadvantage any patient population, a critical requirement for responsible clinical AI deployment.

## Data Availability

The original contributions presented in the study are included in the article/Supplementary material, further inquiries can be directed to the corresponding author.
