# Supplementary figures and images for: Arthralgia and fever as dominant predictors of Chikungunya confirmation: an explainable artificial intelligence approach
Source: Front Med (Lausanne). 2026 Apr 13;13:1814257. doi: 10.3389/fmed.2026.1814257 (PMC13110947; doi:10.3389/fmed.2026.1814257)

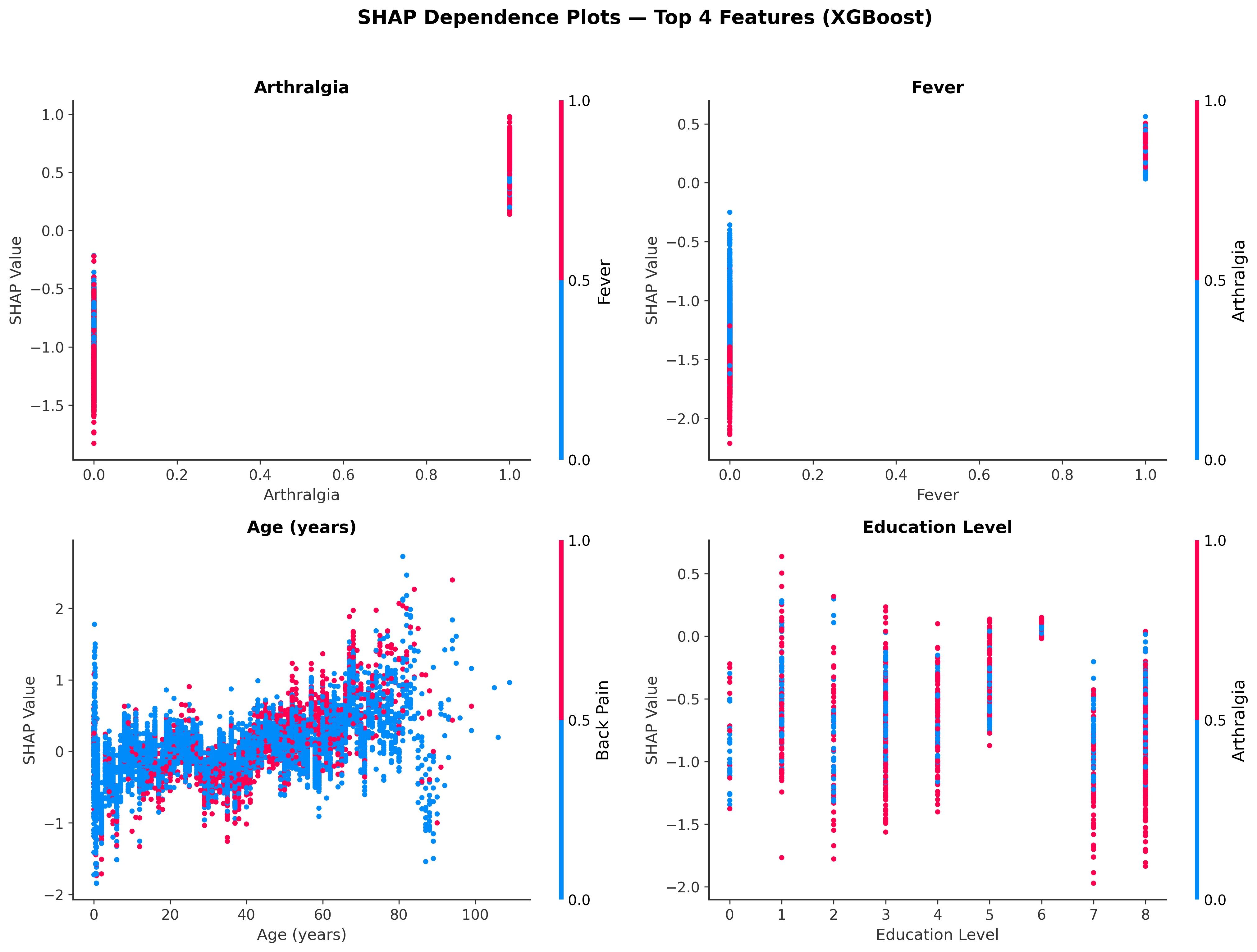

Supplement: Supplementary file 1 [file Data_Sheet_1.zip › SHAP_dependence.png]

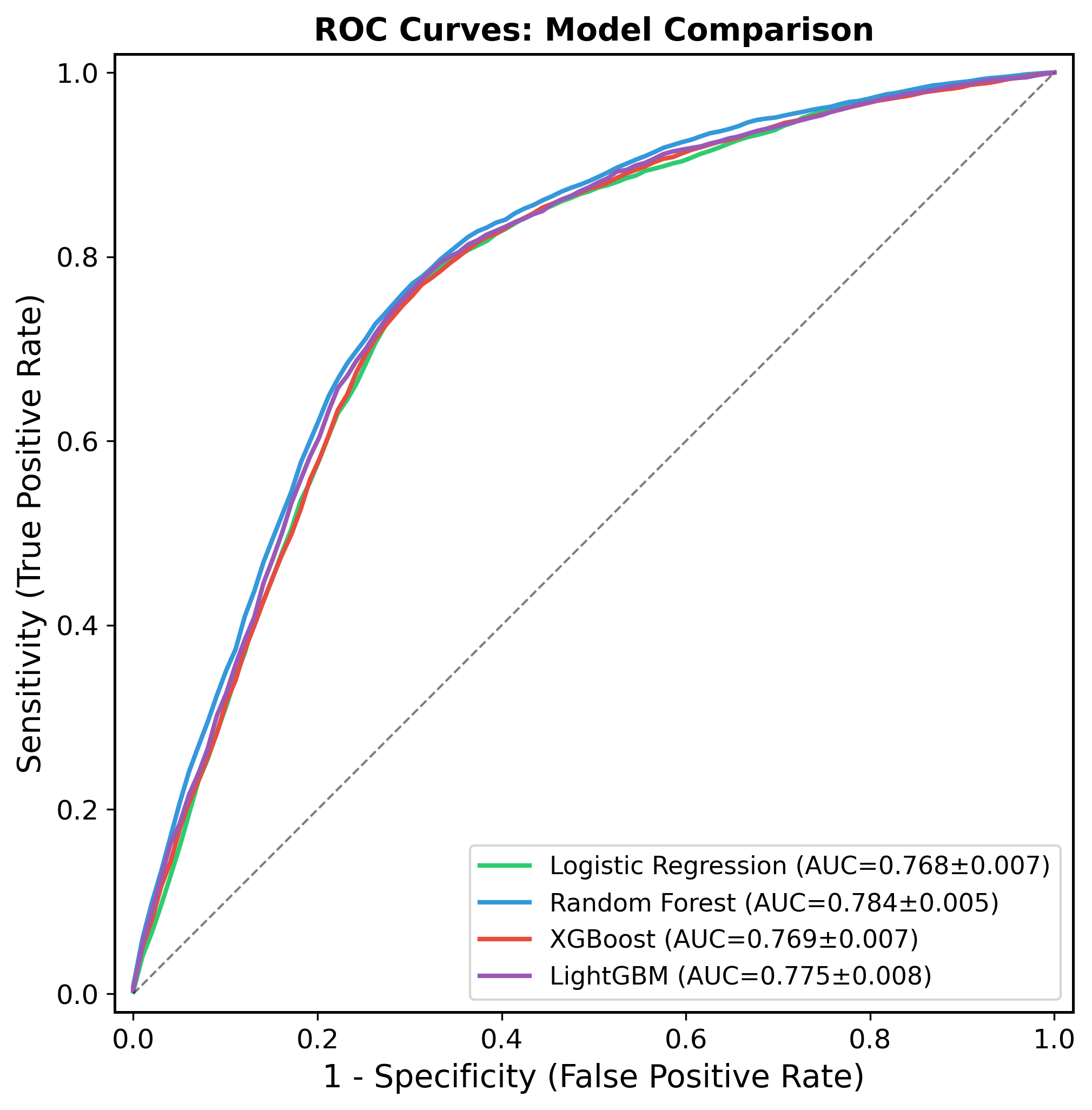

Supplement: Supplementary file 1 [file Data_Sheet_1.zip › ROC_curves.png]

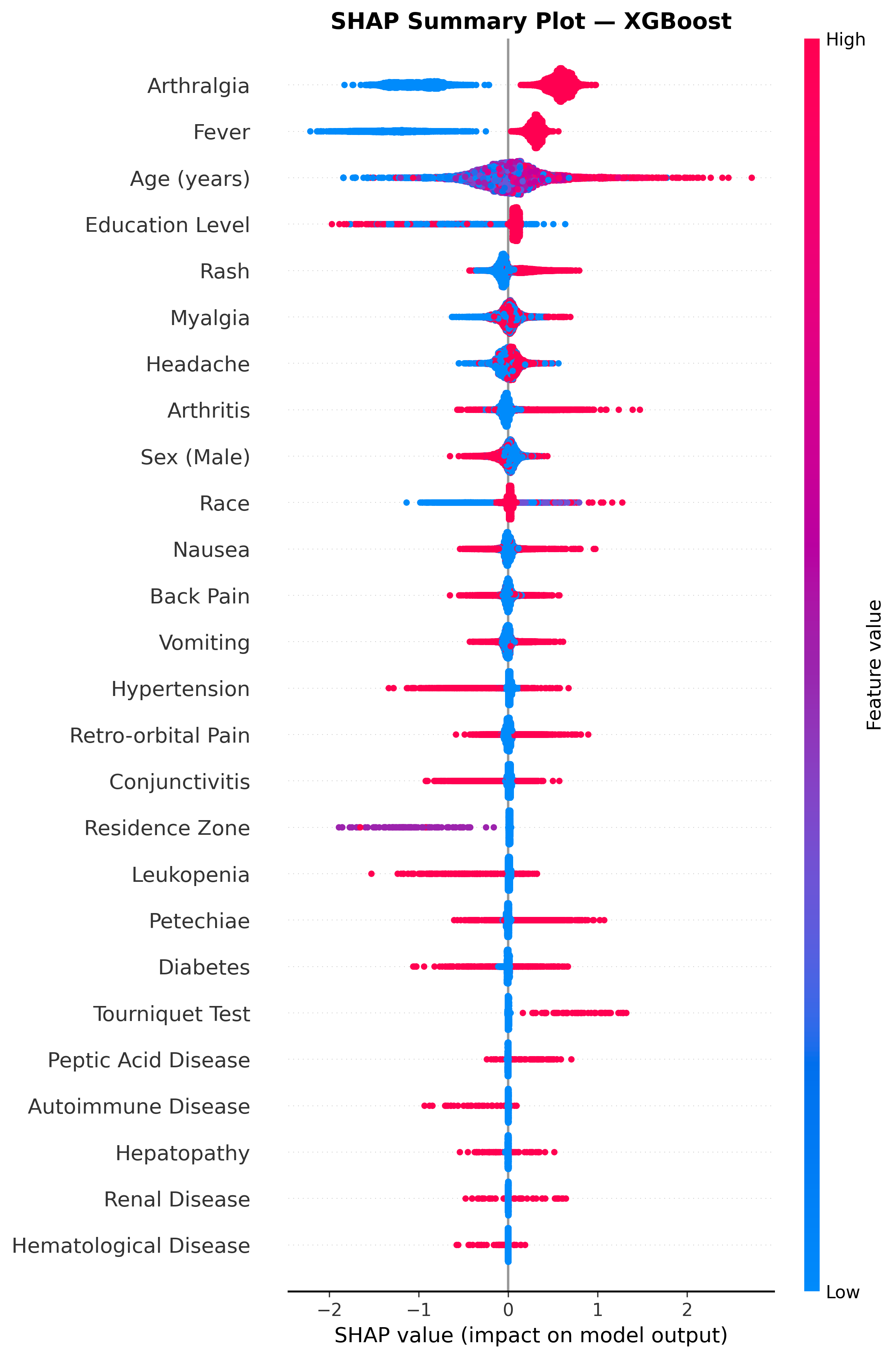

Supplement: Supplementary file 1 [file Data_Sheet_1.zip › SHAP_summary_xgb.png]

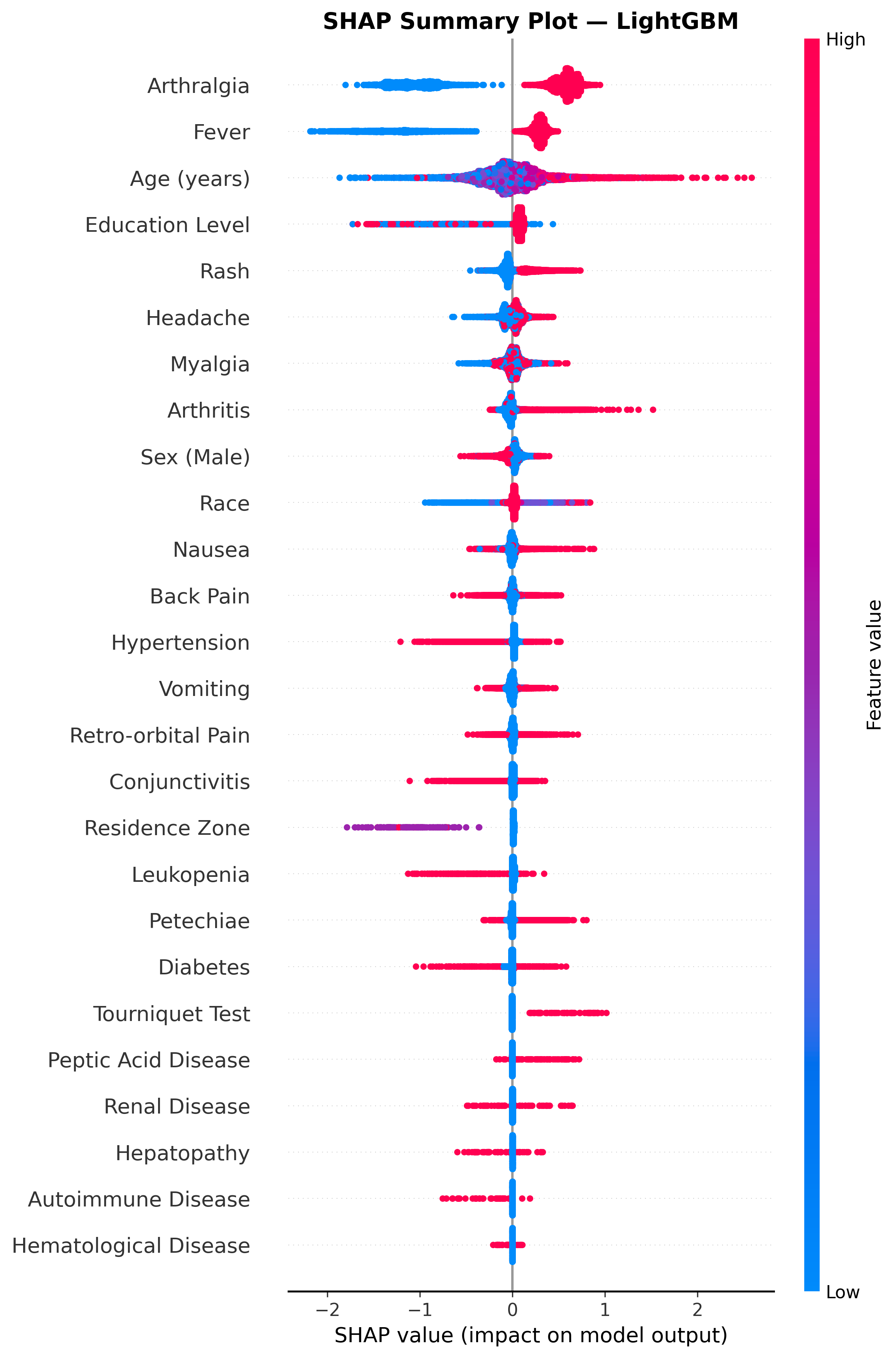

Supplement: Supplementary file 1 [file Data_Sheet_1.zip › SHAP_summary_lgbm.png]

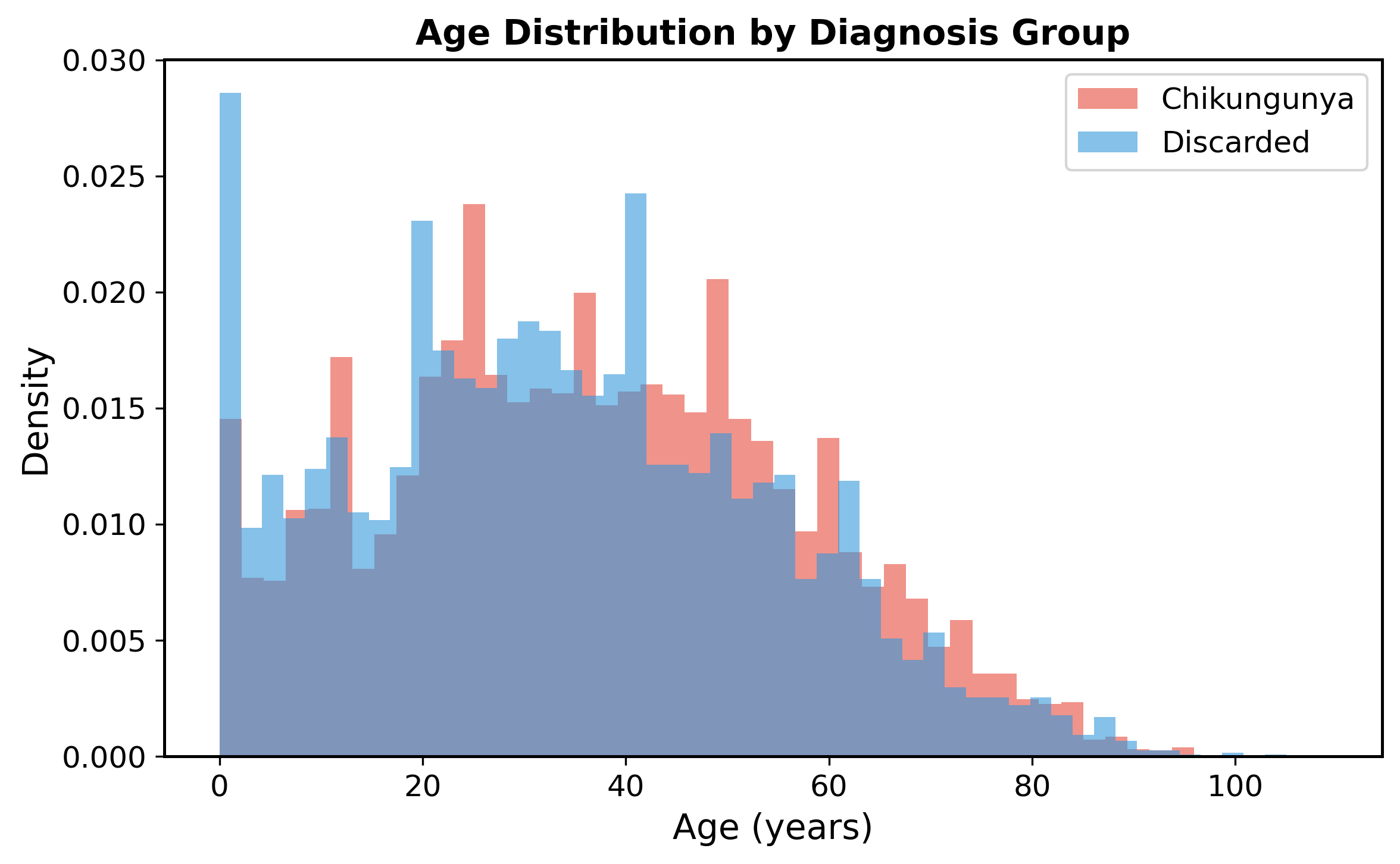

Supplement: Supplementary file 1 [file Data_Sheet_1.zip › age_distribution.png]

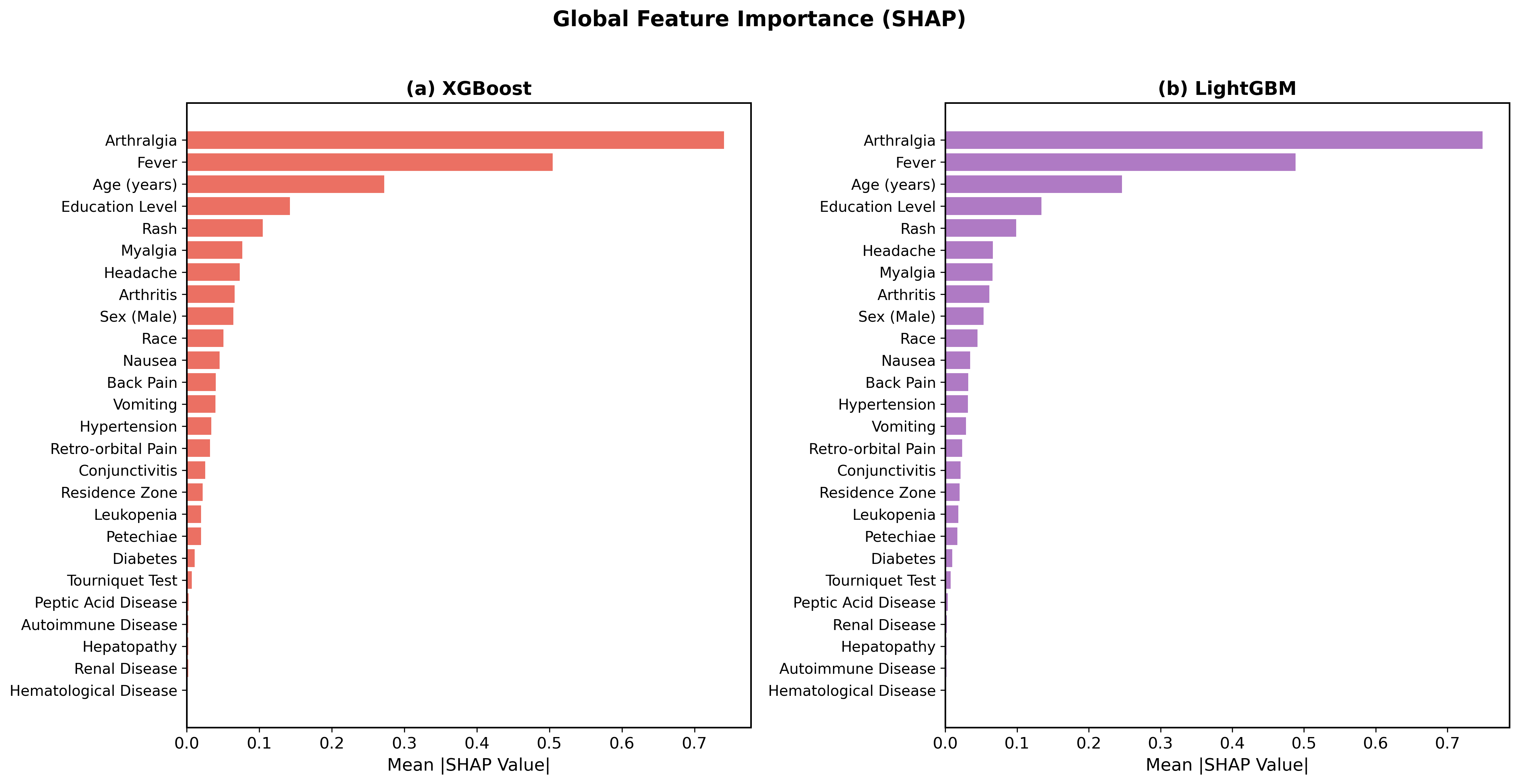

Supplement: Supplementary file 1 [file Data_Sheet_1.zip › SHAP_global_importance.png]

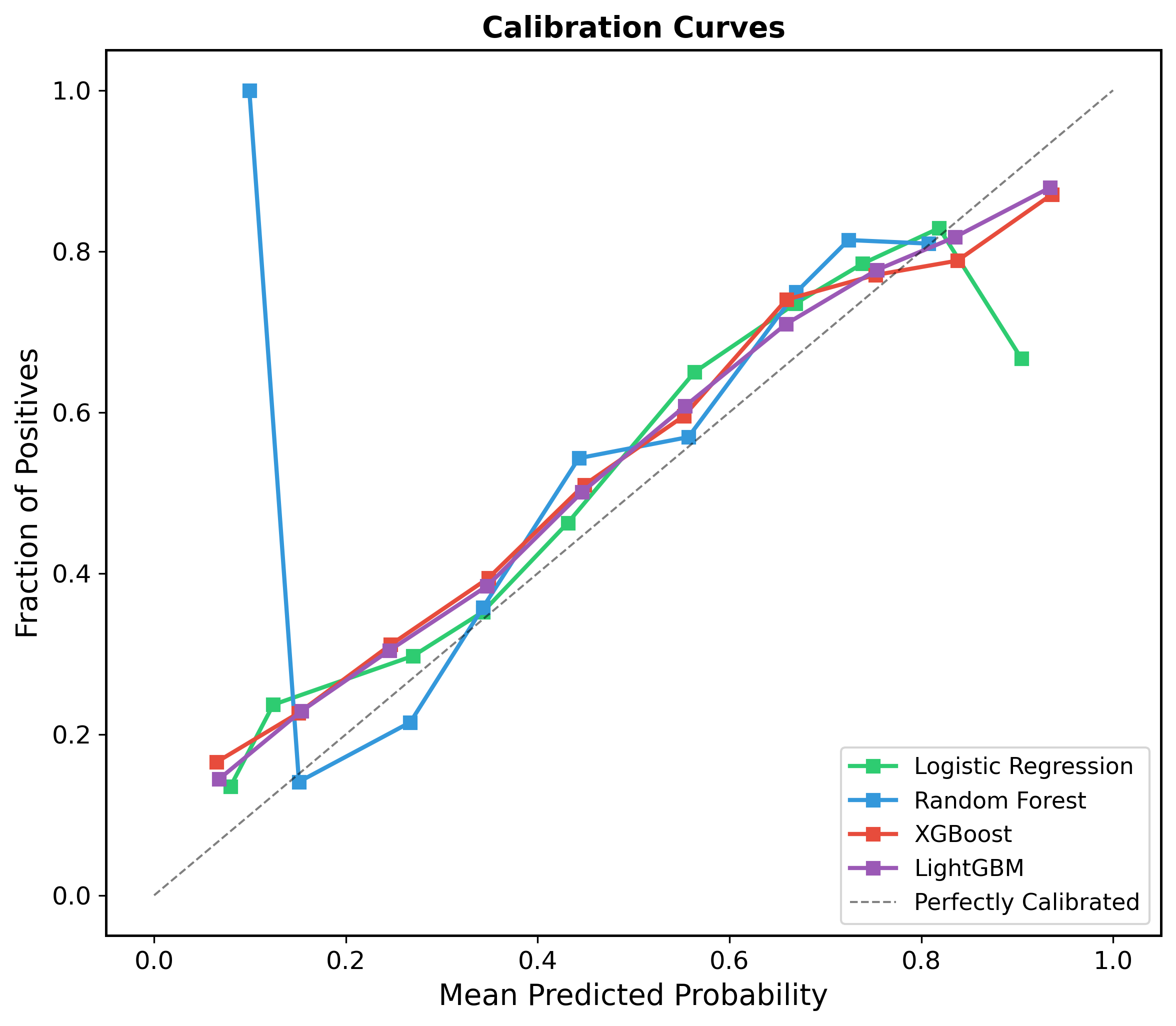

Supplement: Supplementary file 1 [file Data_Sheet_1.zip › calibration_curve.png]

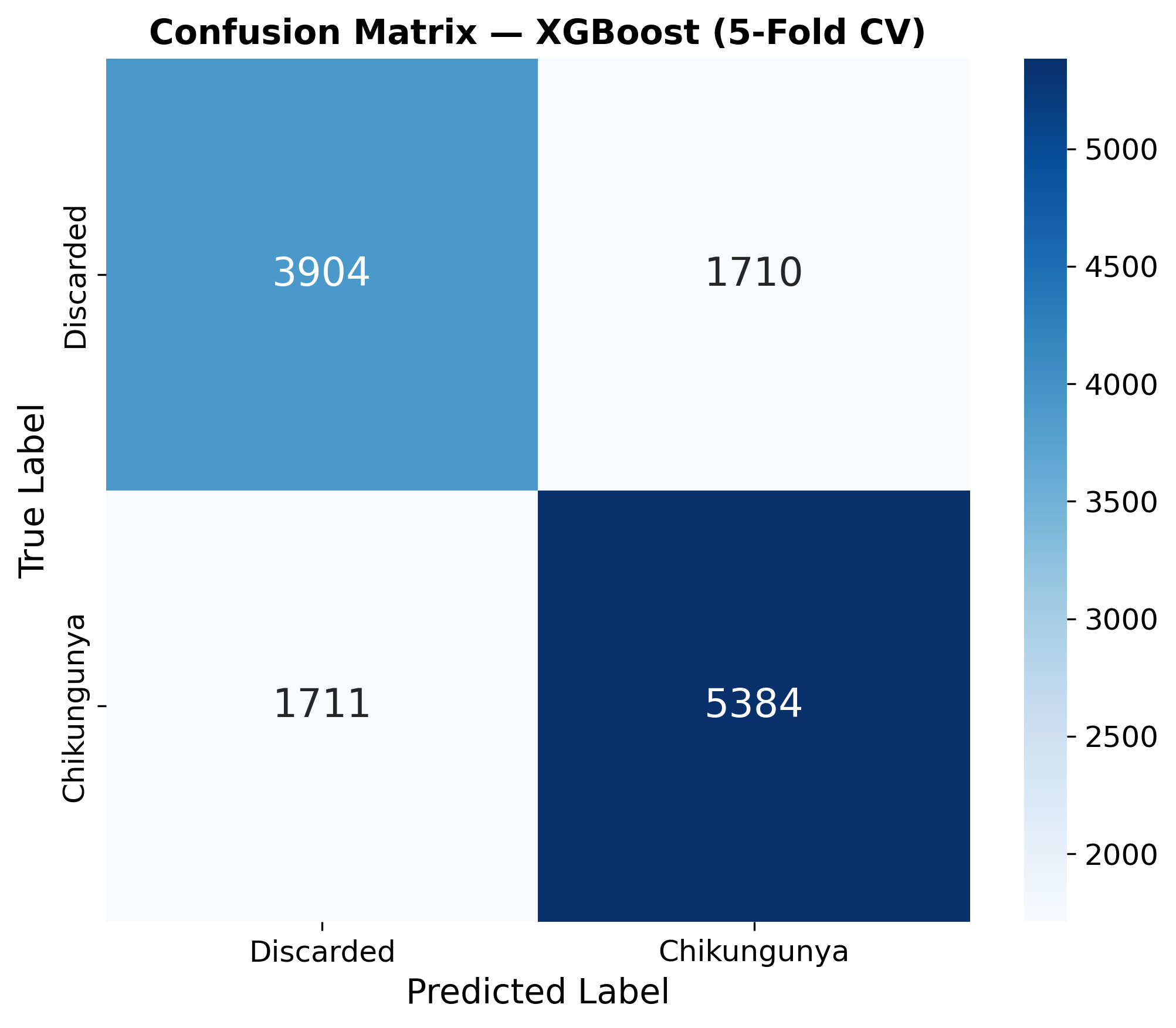

Supplement: Supplementary file 1 [file Data_Sheet_1.zip › confusion_matrix.png]

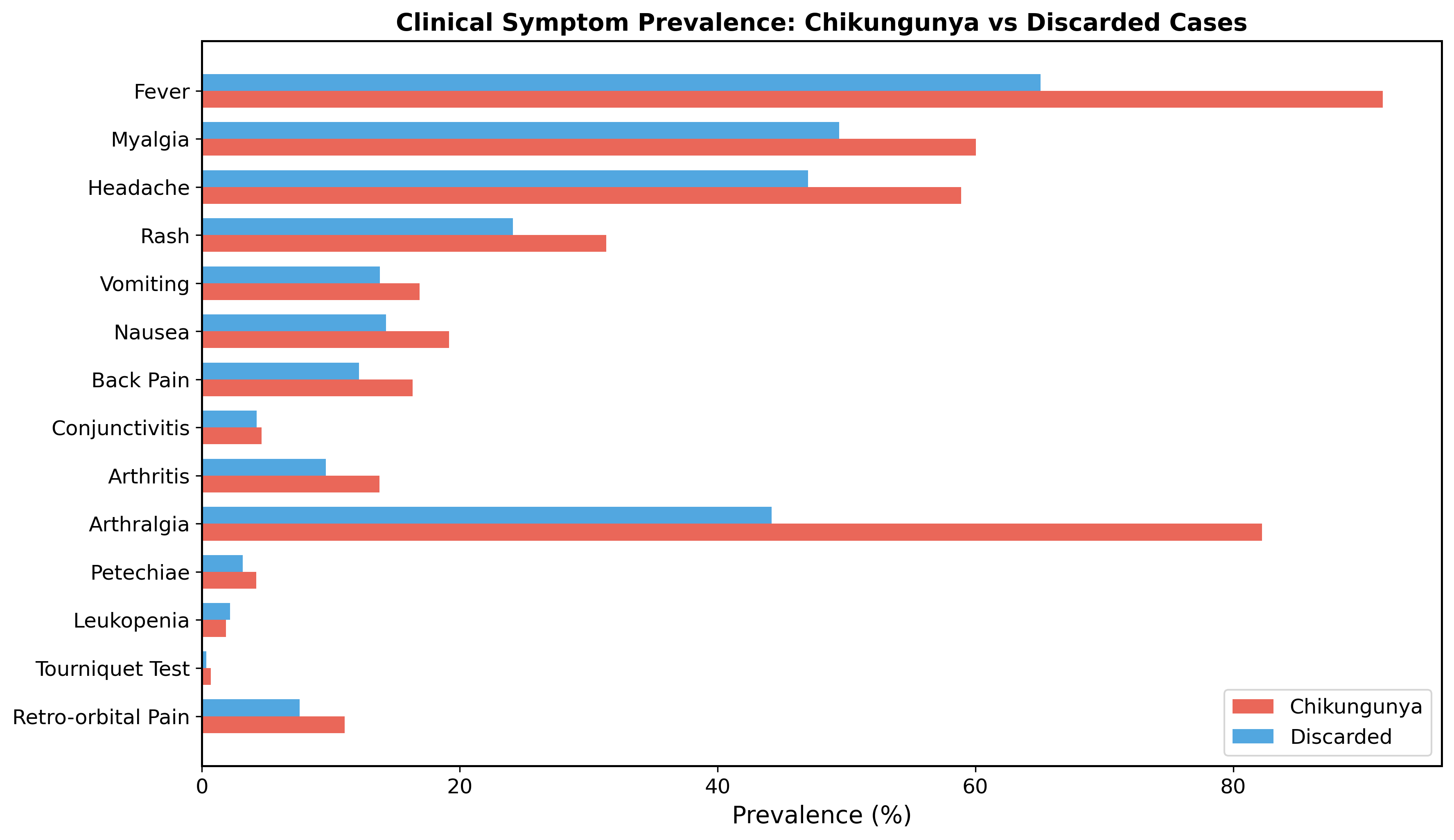

Supplement: Supplementary file 1 [file Data_Sheet_1.zip › symptom_prevalence.png]

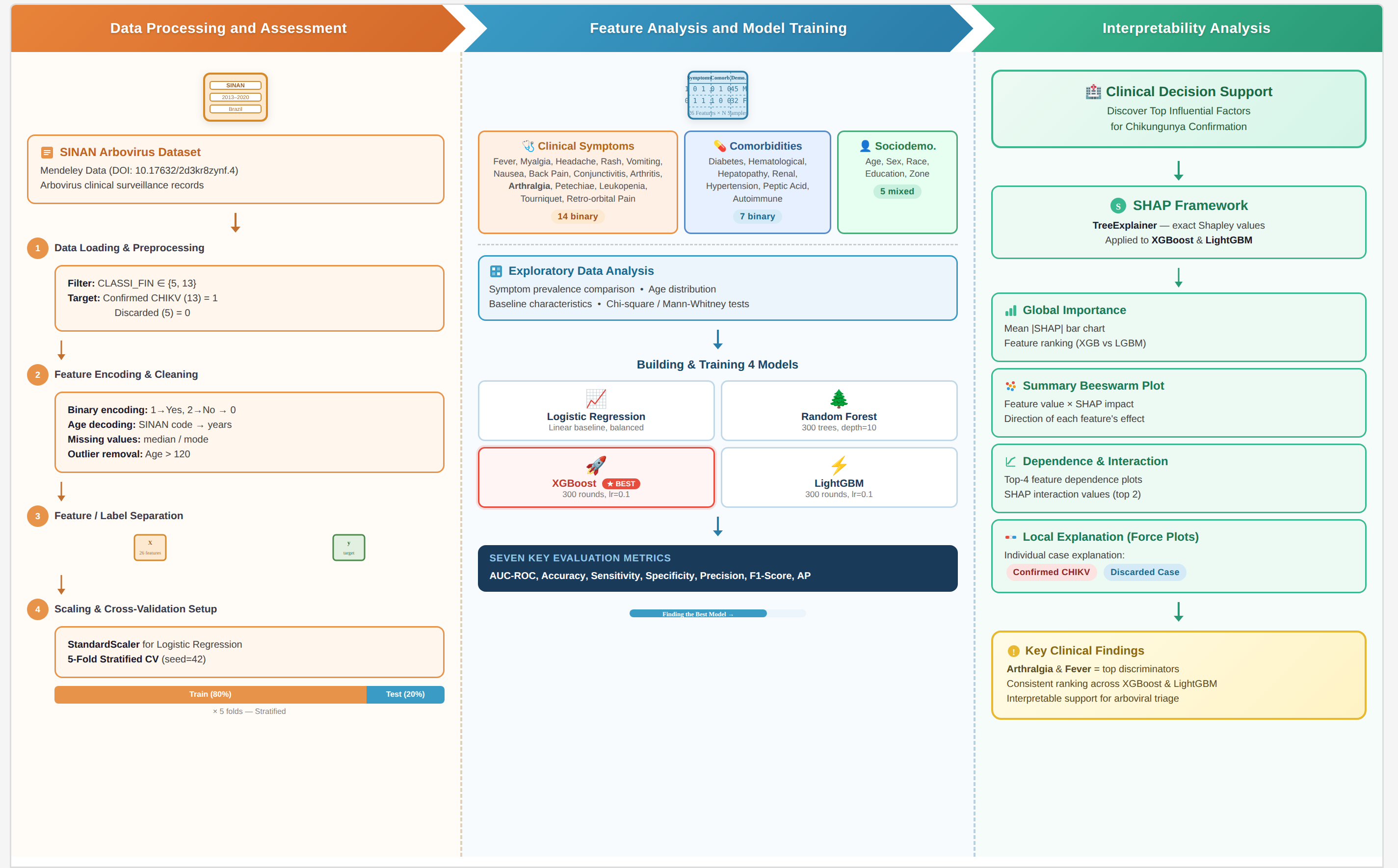

Supplement: Supplementary file 1 [file Data_Sheet_1.zip › workflow_diagram.png]
